# Etiologic Puzzle of Coronary Artery Disease: How Important Is Genetic Component?

**DOI:** 10.3390/life12060865

**Published:** 2022-06-09

**Authors:** Lăcrămioara Ionela Butnariu, Laura Florea, Minerva Codruta Badescu, Elena Țarcă, Irina-Iuliana Costache, Eusebiu Vlad Gorduza

**Affiliations:** 1 Department of Medical Genetics, Faculty of Medicine, “Grigore T. Popa” University of Medicine and Pharmacy, 700115 Iași, Romania; ionela.butnariu@umfiasi.ro (L.I.B.); vgord@mail.com (E.V.G.); 2 Department of Nefrology—Internal Medicine, Faculty of Medicine, “Grigore T. Popa” University of Medicine and Pharmacy, 700115 Iași, Romania; laura.florea@umfiasi.ro; 3 Department of Internal Medicine, “Grigore T. Popa” University of Medicine and Pharmacy, 16 University Street, 700115 Iași, Romania; 4 III Internal Medicine Clinic, “St. Spiridon” County Emergency Clinical Hospital, 1 Independence Boulevard, 700111 Iași, Romania; 5 Department of Surgery II—Pediatric Surgery, “Grigore T. Popa” University of Medicine and Pharmacy, 700115 Iași, Romania; 6 Department of Internal Medicine (Cardiology), “Grigore T. Popa” University of Medicine and Pharmacy, 16 University Street, 700115 Iași, Romania; irina.costache@umfiasi.ro

**Keywords:** coronary artery disease, ischemic heart disease, atherosclerosis, genetic risk factors, heritability, polymorphism, GWAS, PRS

## Abstract

In the modern era, coronary artery disease (CAD) has become the most common form of heart disease and, due to the severity of its clinical manifestations and its acute complications, is a major cause of morbidity and mortality worldwide. The phenotypic variability of CAD is correlated with the complex etiology, multifactorial (caused by the interaction of genetic and environmental factors) but also monogenic. The purpose of this review is to present the genetic factors involved in the etiology of CAD and their relationship to the pathogenic mechanisms of the disease. Method: we analyzed data from the literature, starting with candidate gene-based association studies, then continuing with extensive association studies such as Genome-Wide Association Studies (GWAS) and Whole Exome Sequencing (WES). The results of these studies revealed that the number of genetic factors involved in CAD etiology is impressive. The identification of new genetic factors through GWASs offers new perspectives on understanding the complex pathophysiological mechanisms that determine CAD. In conclusion, deciphering the genetic architecture of CAD by extended genomic analysis (GWAS/WES) will establish new therapeutic targets and lead to the development of new treatments. The identification of individuals at high risk for CAD using polygenic risk scores (PRS) will allow early prophylactic measures and personalized therapy to improve their prognosis.

## 1. Introduction

Despite the remarkable advances made in recent decades in the treatment and prevention of coronary artery disease (CAD), also known as ischemic heart disease (IHD) or coronary heart disease (CHD), it continues to be a major cause of death in industrialized countries. CAD causes more than 3.9 million deaths in Europe, of which 1.8 million in the European Union [1]; in the US, over 18.2 million people have CAD, and annually, more than 805,000 people develope acute coronary syndrome (ACS) (Centers for Disease Control and Prevention, 2019) [2,3]. The risk of CAD and especially acute myocardial infarction (MI) is usually correlated with old age; however, about 5–10% of cases occur before the age of 50. Mortality caused by CAD at a young age has a significant psychological impact on affected families, as well as a substantial negative economic effect. The risk of developing CAD after the age of 40 is about 32% in women and about 49% in men, with women developing CAD a decade later than men due to the protective effect of estrogen hormones, so the onset of the disease follows the onset of menopause [4,5].

The main cause of CAD is the decrease in blood flow in the epicardial coronary arteries caused by obstruction by atherosclerotic plaques (atherosclerotic CAD) [4,6,7]. 

Some studies have shown that the pathophysiological mechanism of CAD is much more complex and, beyond the presence of epicardial atherosclerotic plaques, coronary microcirculation is crucial in the genesis of CAD [8,9].

Atherosclerotic CAD comprises a wide range of clinical features that include asymptomatic subclinical atherosclerosis and its complications such as angina pectoris (PA), acute myocardial infarction (MI) and sudden cardiac death (SCD) [6]. The etiology of CAD is extremely heterogeneous and, although cases of atherosclerotic CAD with monogenic etiology are described, CAD is considered to have a complex multifactorial etiology, being the consequence of the interaction between genetic factors and many environmental factors (diet, physical activity, smoking, other comorbidities) [9,10,11].

Numerous epidemiological studies have been conducted in recent years in an attempt to determine which are the risk factors for CAD, in order to develop models that would allow the development of risk scores (CAD risk prediction models) [4,12,13].

**Figure 1:**
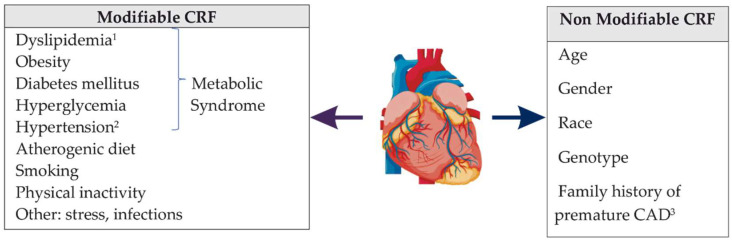
Classification of Cardiovascular Risk Factors (CRF) [4,5,6,7,8,12,13,14,15]. ^1^ Dyslipidemia (total cholesterol > 200 mg/dL; LDL-C > 130 mg/dL; HDL-C < 40 mg/dL; TG > 150 mg/dL) [7]; ^2^ Hypertension is defined as a blood pressure (BP) ≥ 140/90 mm Hg by the European guidelines [8], whereas a lower threshold of BP ≥ 130/80 mm Hg is used by the American guidelines [11]; ^3^ Family history of premature CAD (CAD in male first–degree relative < 55 years, or CAD in female first-degree relative < 65 years) [4,5,6,12,13,14,15].

The Framingham Heart Study (FHS, 1948) was the first study to attempt to elucidate cardiovascular disease risk factors (CRF), followed by the FINRISK study (Finland, 1972) and other cohort studies conducted at Uppsala University in Sweden (ULSAM, PIVUS, POEM, EpiHealth and SCAPIS), INTERHEART study (a case–control study of acute myocardial infarction in 52 countries) and another study in New Zealand (PREDICT Cardiovascular Disease Cohort). In these studies, CAD risk factors are classified into two categories: modifiable risk factors (hypercholesterolemia, smoking, diabetes mellitus, systolic hypertension, sedentary lifestyle) and non-modifiable risk factors (age, sex, family history for CAD) (Figure 1) [4,5,6,7,8,12,13,14,15].

Currently, the Multi Ethnic Study Group of Atherosclerosis (MESA) is considering the inclusion of coronary artery calcification (CAC) in the category of risk factors for CAD [13]. The European Association for Cardiovascular Prevention and Rehabilitation (EACRP) and the American College of Cardiology/American Heart Association (ACC/AHA) have developed practical guidelines, based on CAD risk prediction models, to reduce the risk of acute coronary syndromes (ACS) [13]. 

The phenotypic variability in CAD patients is most likely due to complex pathophysiological mechanisms involving numerous interactions between different genetic factors encoding different molecules, as well as their interactions with environmental factors. The identification of risk alleles, which determine an increased susceptibility to CAD, is of great interest. Geneticists point to the need to achieve polygenic risk scores (PRS) that could play an important role in identifying people at high risk for CAD. In their case, the early implementation of prophylactic measures would help to improve the prognosis and life expectancy [6,13]. 

The aim of our paper is to provide an in-depth analysis of the data available in the literature on the role of genetic factors in the etiology of CAD. We also focused on the complex interaction between genetic and environmental factors. Thus, we performed the most comprehensive analysis of the methods used in the study of genetic factors involved in the etiology of CAD and their results. We highlighted the advantages and limitations of each type of study, as well as the perspectives that these data offer for the implementation of effective prophylactic measures, early diagnosis in order to avoid lethal complications (MI, SCD), personalized therapy, and last but not least, for the development of innovative drugs.

## 2. Literature Search Strategies and Data Collection

The data synthesized and presented in this review was obtained by examining the literature (Google Scholar, PubMed, MEDLINE, OMIM, MedGen databases) and using the following keywords: ischemic heart disease (IHD), coronary artery disease (CAD), atherosclerosis, genetic risk factors, heritability, monogenic CAD/IHD/CHD, polygenic CAD/IHD/CHD, candidate gene-based association studies (CGS), Linkage-studies (LS), Genetic linkage-analysis (LA), Genome-wide association studies (GWAS) or Whole Exome Sequencing (WES) (Table 1).

## 3. Phenotypic Variability of Coronary Artery Disease

The study of genetic factors involved in the pathogenesis of CAD has proven to be a difficult task due to phenotypic variability which is closely correlated with genetic heterogeneity. However, in recent years, due to the development of molecular technology, significant progress has been made in the genetic and genomic studies of CAD and MI [6].

Coronary atherosclerosis is the main pathogenic mechanism in CAD, regardless of its clinical form, that ranges from subclinical atherosclerosis to chronic and acute coronary sindroms and SCD [6,14]. Some data indicate that about 50% of SCD are due to MI, and about a third of MI cases are silent [6,13]. The average age at which the first MI occurs varies between the two sexes, being 64.9 years for men and 72.3 years for women [1]. In the case of octogenarian patients, a history of atherosclerotic CAD is usually present, often preceded by a long asymptomatic period, but episodes of MI are also present in young adults (<40 years), in the absence of a positive history of coronary atherosclerosis [6].

The formation of atherosclerotic plaque on the wall of the coronary arteries is a chronic process, with an early onset, and is initiated/favored by chronic endothelial lesions. Plaques progress, causing vascular obstruction and tissue ischemia over time. Rupture or ulceration of unstable plaques causes ACS, such as unstable angina, acute myocardial infarction (MI), and sudden cardiac death (SCD). Rupture of a vulnerable atherosclerotic plaque, accumulation and activation of platelets, fibrin deposition, thrombus formation and possible occlusion of vessels are the pathophysiological mechanism of MI [6,9].

The formation and progression of atherosclerotic plaques involves many biochemical processes involving enzymes, receptors and their ligands, molecules that are encoded by various genes that interact with environmental factors. Thus, lipid and apolipoprotein metabolism, inflammatory response, endothelial function, platelet function, thrombosis, homocysteine metabolism, insulin sensitivity and blood pressure regulating mechanism may be disrupted [5,6].

The location of atherosclerotic stenosis in the coronary vessels and may reflect how genetic variability may influence the production of atherosclerosis under certain conditions of dynamic blood flow. Isolated aorto-ostial stenosis (left or right main coronary artery ostium) and bifurcation lesions are more evident in relation to turbulent flow and endothelial response to flow dynamics. In some studies in mice, *PECAM-1* gene polymorphism has been shown to be an important factor in the atherogenic changes seen in ApoE-deficient mice, the effects of which are dependent on the location of atherosclerosis in the coronary arteries. These results suggest that the heterogeneity of atherosclerosis localization could be influenced by certain polymorphisms of genes involved in the process of atherosclerosis, under different dynamic flow conditions [6,140]. On the other hand, diffuse atherosclerosis is more commonly seen in patients with diabetes mellitus (DM) [5,6,7].

## 4. Heritability of Atherosclerotic CAD

The importance of genetic factors in the etiology of CAD and MI has been demonstrated by numerous clinical and population studies. Several cohort studies have shown that the family history of CAD is associated with an increased risk of the disease, with family aggregation being reported since the middle of the last century [6,141]. The heritability of CAD is estimated to be between 40–60%, according to family-based association studies, those of twin studies or GWASs [6,140]. Subsequently, a significant association of cases with early-onset CAD was demonstrated in patients who had first-degree relatives with early-onset CAD [142].

The Framingham Heart Study (FHS) later confirmed that a family history of premature CAD (defined as the presence of an affected first-degree relative, male under 55 or female under 65) is an independent risk factor for CAD [143]. The importance of genetic factors is confirmed by the association between the increased number of affected relatives and the lower age of onset. Family-based studies have shown that a positive family history for CAD increases a person’s risk of developing the disease two to seven times compared with a person with no family history of the disease. In fact, studies based on the results of coronary angiography have indicated that the etiology of CAD with a positive family history is independent of environmental factors (atherogenic diet, obesity, hypertension, smoking or alcohol consumption) [6,144]. 

Other population studies (the Western Collaborative Group Study, British Regional Heart Study, German PROCAM study) confirmed the strong independent association between the positive family history for CAD and the risk of CAD and MI in offspring [6].

Twin studies have provided important information on the genetic component of CAD. Thus, in monozygotic (MZ) twins a concordance was observed regarding early-onset CAD and the involvement of the same coronary arteries [4,6]. Data from the Swedish Twin Registry included 20,966 twins, who were followed for 36 years, indicate that if one of the twins died by CAD, the twin brother’s risk of developing lethal CAD is 8.1 for monozygotic (MZ) twins and 3.8 for dizygotic (DZ) twin. The estimated heritability of CAD differs between the two sexes, being estimated at 57% in males and 38% in females and the influence of genetic factors was in the age range between 36 and 86 years [145]. 

A prospective analysis of 8000 pairs of twins included in the Danish Twin Registry showed an increased incidence of CAD deaths in MZ twins compared with DZ twins (44% vs. 14%), with a heritability of mortality due to heart disease (heritability of frailty, liability to death) estimated at 0.53 in men and 0.58 in women [146]. 

Genetic factors act independently of the environmental factors involved in the etiology of CAD, but the variable phenotype results from their permanent interaction. The study of monogenic mutations that cause atherosclerotic CAD as well as polygenic risk factors (genetic polymorphisms) was performed by two types of studies: linkage analysis (LA) and association studies (CGS, GWAS and WES) [4,7,8].

*Genetic Linkage analysis* (*LA*) *studies* investigate the cosegregation (association with the onset of disease) of polymorphic DNA markers distributed throughout the genome with hereditary transmission of the disease in that family and aims to detect the genomic region where the gene is located, identifying the disease-causing gene variant. It has been used successfully in identifying monogenic mutations that cause disease and, less so in the case of polygenic diseases, given the complexity of their etiology [4,5,6,9,10].

*Association studies* are an alternative method of studying polygenic inheritance and usually use the candidate-gene approach (CGA). Starting from the known pathophysiological mechanisms and from the genes that are supposed to intervene in different stages, the hypothesis of the association of these genes with the respective disease is analyzed/tested. In the case of CAD, the genes involved in lipid metabolism, lipopoteins, DM, and hypertension were analyzed. However, this approach is limited by incomplete knowledge of the pathogenic mechanisms of CAD. The advances in the last 20 years in molecular technology have allowed extensive analysis of the whole genome (GWAS) or exome (WES) providing important information on the genetic causes of diseases with complex, multifactorial etiology [4,7,10].

## 5. Monogenic Etiology of CAD/MI

Candidate gene-based association studies (CGS) and linkage analysis (LA) studies have identified many genes whose mutations cause rare monogenic forms of CAD, most of which are involved in lipid metabolism (atherosclerotic CAD) (Table 1).

### 5.1. Monogenic Lipid Disorders

#### 5.1.1. Genetic Causes of Elevated Plasma LDL-Cholesterol Level

Atherosclerosis and coronary artery obstruction are the leading causes of CAD, and low-density lipoprotein cholesterol (LDL-C) molecules, in particular, contribute to the development and progression of atherosclerotic plaque. Considering this, mutations of genes involved in lipid metabolism that increase plasma levels of LDL-C have an important contribution to CAD [4,5,6]. 

Familial Hypercholesterolemia type 1 (FH)

Mutations of genes encoding the LDL receptor (LDLR), apolipoprotein B-100 (ApoB-100), an LDL-C receptor ligand, and proprotein convertase, subtilisin/kexin-type 9 (PCSK9) cause familial hypercholesterolemia type 1 (FH), (OMIM, 606,945), an autosomal dominant disorder [16].

*a*. *Low-Density Lipoprotein Receptor (LDLR) Gene*

The *LDLR* gene (located on chromosome 19p13.2) encodes an 860 amino acid protein (LDLR) that is involved in the absorption and degradation of LDL-C. *LDLR* gene mutations cause about 85–90% of FH cases and are associated with abnormal or dysfunctional LDLR. There are currently 3839 known mutations that are distributed throughout the *LDLR* gene [4,5,6]. 

Homozygous mutations in the *LDLR* gene cause early-onset CAD in childhood and are characterized by plasma LDL-C levels approximately 6–10 times higher than normal (600–1200 mg/dL), detected at birth. Extremely high LDL-C levels can cause the first MI to occur before the age of 20, and in the absence of treatment, death occurs before the age of 30. Approximately 5% of people with CAD and MI before the age of 60 have heterozygous mutations of the *LDLR* gene, with 2 times higher plasma levels of LDL-C (300–400 mg/dL) being detected at birth. Among heterozygotes, 75% of men and 45% of women develop CAD by the age of 60, and 50% of men and 15% of women die of MI by this age [17]. 

In familial cases of CAD, the genetic testing algorithm is based on the initial investigation of the *LDLR* gene, and in case of a negative result the mutations of the *ApoB-100* and *PCSK9* genes are tested [18].

*b*. *APOB Gene*

The *APOB* gene (located on chromosome 2p24.1) encodes ApoB-100 apolipoproteins, the main protein component of apolipoproteins synthesized in the liver: chylomicrons (CM), very low-density lipoprotein (VLDL) and low-density lipoprotein (LDL), and ApoB-48 (synthesized exclusively in the intestine). ApoB-100 has an LDLR binding domain, helping to regulate plasma cholesterol levels, removing LDL-C from the body, and binding to heparin and various proteoglycans in arterial walls [4,19]. 

Two allelic variants of the *APOB* gene: C10580G (p.Arg3527Gln) [20] and C10800T (p.Arg3531Cys) [21] have been associated with decreased affinity for LDLR, leading to familial hypercholesterolemia type 2 (FHCL2) (OMIM 144,010) also called familial defective apolipoprotein B-100 (FDB), an autosomal dominant genetic disorder associated with hyperlipidemia and increased risk of early atherosclerosis [16].

In a study that combined genetic linkage analysis (LA) with WES in a family members with FH (familial hypercholesterolemia type 1, AD), Thomas et al. [22] identified a third mutation (p.Arg50Trp) in exon 3 of the *APOB* gene [22]. 

Other mutations of the *APOB* gene cause hypobetalipoproteinemia (FHBL) (OMIM, 615,558) and abetalipoproteinemia (ABL) (OMIM, 200,100), two rare, autosomal recessive conditions characterized by hypocholesterolemia and malabsorption of lipid-soluble vitamins, which causes retinal degeneration, neuropathy and coagulopathy [16].

*c*. *PCSK9 Gene*

The *PCSK9* gene (proprotein convertase, subtilisin/kexin-type, 9, located on chromosome 1p32.3) encodes neural apoptosis-regulated convertase 1 (NARC 1), a serum protease that reduces both hepatic and extrahepatic LDLR levels, causing increase plasma LDL-C level [23]. Functional mutations in the *PCSK9* gene cause autosomal dominant familial hypercholesterolemia-3 (FHCL3, FH3, HCHOLA3) (OMIM, 603,776) [16].

Initially, nine types of mutations in the *PCSK9* gene were reported in families whose members had a form of autosomal dominant transmitted hypercholesterolemia [24]. These mutations are associated with a decrease in the number of LDLR, the consequence being the increase plasma total cholesterol (TC) levels and LDL-C with the appearance of tendon xanthomas, premature CAD, MI and ischemic stroke. A GWAS study indicated that the single nucleotide polymorphism (SNP) rs11206510 (risk allele T) located on *PCSK9* gene was associated with an increased risk of CAD and MI [25].

The *PCSK9* gene polymorphism is correlated with both plasma lipid levels and the response to lipid-lowering drugs (statins). Studies in patients with hypocholesterolemia (Atherosclerosis Risk in Communities-ARIC and Dallas Heart Study) have found that loss-of-function mutation of *PCSK9* gene have a protective effect against CAD and MI [26].

In a meta-analysis by Chuan et al. [27], the association between the allelic variant rs562556 (c.1420G>A, I474V) located in exon 9 of the *PCSK9* gene and the low plasma total cholesterol (TC) levels and LDL-C was highlighted [27]. These findings have led to the development of *PCSK9* inhibitors as new agents to reduce plasma cholesterol levels [27].

*d*. *LDLRAP1 Gene*

The loss-of-function mutation in the *LDLRAP1* (LDL receptor adapter protein 1) gene (located on chromosome 1p34-1p35) are extremely rare and lead to the appearance of a truncated or non-functional LDLRAP1 protein (required for internalizing LDLR into hepatocytes). Compound homozygous or heterozygous individuals for pathogenic mutations of *LDLRAP1* show an autosomal recessive form of hypercholesterolemia (ARH/FHCL4) (OMIM, 603813) [16]. ARH can be considered a phenocopy of homozygous familial hypercholesterolemia, which progresses with increased risk for atherosclerotic CAD (with rapid fatal evolution, despite conventional therapies) and, aortic valve stenosis. Lomitapide combined with conventional drugs that reduce plasma LDL-C levels appears to be an effective treatment for ARH [28].

The literature describes 50 cases of ARH, in patients of Mediterranean or Middle Eastern origin. In a study by Arca et al. [29] which included 28 people from 17 unrelated families in Sardinia, two types of mutations of *LDLRAP1* gene were identified: a frameshift mutation C432insA (p.FS170stop) in exon 4 (ARH1) and a nonsense mutation C65G->A (p.Trp22ter) in exon 1 (ARH2). In three of the cases, a compound heterozygous genotype was detected as a result of the ancient recombination of the two mutations ARH1 and ARH2 [29]. Only four of the reported cases had the homozygous genotype ARH1, the patients coming from the Italian mainland [29].

#### 5.1.2. Genetic Causes of Low Plasma HDL-Cholesterol Level

Prospective cohort studies revealed that low high-density lipoprotein-cholesterol (HDL-C) (HDL-C < 35–40 mg/dL according to current guidelines or age-and sex-adjusted plasma HDL-C concentration below the 10th percentile) is a significant, negative risk factor, independent of traditional CAD risk factors [30]. In 40% of CAD cases, low plasma HDL-C levels are detected, and the genetic etiology is incriminated in a proportion of 40–70% [4].

*a*. *APOA1 Gene*

The *APOA1* gene is located on chromosome 11q23.3 and encodes the apolipoprotein AI (ApoA-I), a component of HDL-C, with a major role in its metabolism. ApoA-I acts as a cofactor for the enzyme LCAT (lecithin-cholesterol acyltransferase), which is responsible for removing cholesterol from tissues and plasma through a cholesterol esterification process. Compound homozygous, heterozygous, and heterozygous mutations in the *APOA1* gene cause familial hypoalphalipoproteinemia (Hypoalphalipoproteinemia, Primary, 2) (OMIM, 618,463), which also includes the Combined Apo-I and apoC-III, both being autosomal recessive diseases [4,16,31].

The *APOA1* gene polymorphism is associated with low plasma HDL-C levels and an increased risk of premature CAD. Homozygous mutations cause complete absence of ApoA-I, with low HDL-C < 5 mg/dL and normal plasma LDL-C and TG levels. Missense mutations in the *APOA1* gene (which are almost always heterozygous) affect the structure of the ApoA-I protein, often causing impairment of its function, associated with low plasma levels of ApoA-I and HDL-C [4,31].

Yamakawa-Kobayashi et al. [32] analyzed the polymorphism of the *APOA1* gene in a group of 67 Japanese children with low plasma HDL-C levels, identifying four different mutations (3 frameshifts mutation and 1 splice site mutation) [32]. In their case, the plasma levels of ApoA-I were reduced by approximately 50% of the normal value (below the first percentile of the general population distribution) (80 mg/dL). The frequency of hypoalphalipoproteinemia due to *APOA1* gene mutations in the Japanese population was estimated at 0.3% in the general population and 6% of individuals with low plasma HDL-C levels [32].

In a Danish study, Haase et al. [33] showed that certain rare allelic variants (eg variant A164S) of the *APOA1* gene are associated with decreased plasma levels of ApoA-I and HDL-C and predispose to amyloidosis, with an increased risk of CAD and MI [33]. The ApoA-I(Milano) and ApoA-I(Paris) variants are rare cysteine variants of ApoA-I which, although they cause decreased plasma HDL-C levels and increased plasma TG levels, have a cardioprotective effect. They can also produce a HDL-C deficiency in the absence of cardiovascular disease (CVD) [34].

*b*. *ABC1 Gene*

The *ABC1* gene (located on chromosome 9q31.1) encodes ATP-binding cassette transporter 1 (ABCA1) a cellular exporter of cholesterol, which causes the removal of free intracellular cholesterol and phospholipids from extrahepatic cells, having a protective role against CVD. Mutations in the *ABCA1* gene cause loss of transporter protein function and may contribute to the process of atherogenesis present in common inflammatory diseases and metabolic disorders. Compound homozygous or heterozygous mutations in the *ABCA1* gene cause Tangier disease (TGD) (OMIM, 205,400), a rare autosomal recessive disorder characterized by extremely low plasma HDL-C levels (HDL-C < 5 mg/dL) and ApoA- I ≤ 10 mg/dL and, increased risk of early-onset CAD [16].

Clinically, TGD is characterized by yellow-orange pharyngeal tonsils, hepatosplenomegaly, corneal opacity, lymphadenopathy, and peripheral neuropathy [35]. The data show that 331 mutations in the *ABC1* gene are reported in the literature [36]. About 1 in 400 individuals in the general population is a heterozygous carrier of a loss-of-function mutation in the *ABCA1* gene (frameshift, nonsense and splicing mutation). In their case, the plasma levels of HDL-C and ApoA-I are variable, being able to be reduced by up to 50% compared with normal levels [35]. Mokuno et al. [37] showed in a Japanese study that *ABCA1* R219K polymorphism (G1051A, rs2230806) K allele is associated with a higher plasma HDL-C levels that may be protective against the risk of CAD in Asian and Caucasian patients [37].

*c*. *LCAT Gene*

The *LCAT* gene located on chromosome 16q22.1 encodes the enzyme lecithin-cholesterol acyltransferase (LCAT) which is involved in removing cholesterol from the blood and tissues. LCAT catalyzes the esterification of free cholesterol with acyl groups derived from lecithin, an essential step in the maturation of HDL-C. The enzyme LCAT has two major activities: alpha-LCAT activity, which helps attach cholesterol to high-density lipoprotein (HDL), and beta-LCAT activity, which facilitates the attachment of cholesterol to other lipoproteins (VLDL and LDL) [38]. Compound homozygous or heterozygous mutations in the *LCAT* gene cause LCAT enzyme deficiency, and two entities are described: Norum disease (OMIM 245,900) and fish-eye disease (FED/Partial LCAT deficiency) (OMIM 136,120) [16].

Norum disease is a rare autosomal recessive condition, characterized by the presence of corneal opacity, hemolytic anemia, proteinuria, renal failure and atherosclerosis. In Norum disease both activities (alpha and beta) of the LCAT enzyme are lost causing very low levels of HDL-C (below the 5th percentile), increased TG and decreased LDL-C [39]. In the FED, only alpha-LCAT activity is lost, beta activity being preserved, allowing the esterification of cholesterol in VLDL and LDL-C, but not in HDL-C [10].

There are few longitudinal follow-up studies of molecular defects associated with LCAT deficiency syndromes. A total of 138 *LCAT* gene mutations were identified, mostly in exons 1 and 4, without a correlation between genotype and phenotype or ethnicity. 

It has been observed that there is a significantly higher risk of CAD in the FED compared with Norum disease [38]. This observation is clinically important, suggesting that management must be customized according to the LCAT deficiency phenotype. Thus, in Norum disease the priority is to improve both CVD and progression to end-stage renal disease (ESRD), whereas in FED patients the priority is to reduce cardiovascular risk [38].

#### 5.1.3. Genetic Etiology of Hypertriglyceridemia

Many studies have shown that higher plasma TG levels are a strong independent risk factor for CAD [40]. Among the different types of lipoproteins, chylomicrons (CM) and VLDL particles are the main carriers of TG, whereas LDL-C and HDL-C are mainly involved in cholesterol transport. Plasma TG levels are also influenced by environmental factors (diet, lifestyle, sedentary lifestyle, smoking) [40].

*a*. *LPL Gene*

The *LPL* gene (located on chromosome 8p21.3) encodes lipoprotein lipase (LPL), an enzyme that limits the rate at which VLDL convert to LDL-C. Compound homozygous or heterozygous mutations in the *LPL* gene cause Lipoprotein lipase deficiency (LPL deficiency or type I hyperlipoproteinemia) (OMIM, 238,600), a rare autosomal recessive condition [16]. The disease is characterized by an extremely elevated serum triglyceride level, lactescent serum (milky creamy serum), decreased plasma HDL-C and LDL-C levels, eruptive xanthomas, acute abdominal pain, hepatosplenomegaly, and sometimes early-onset atherosclerotic CAD. Heterozygous individuals may have mild hyperlipidemia and reduced postheparin plasma lipolytic activity (PHLA), and early atherosclerosis does not appear to be a feature. It is estimated that approximately 20% of patients with hypertriglyceridemia carry only six common *LPL* gene mutations (Asp9Asn, Asn291Ser, Trp86Arg, Gly188Glu, Pro207Leu, Asp250Asn) associated with type I hyperlipoproteinemia. Testing for these mutations is especially recommended in patients at high risk for premature atherosclerosis. The S447X polymorphism (SX genotype and X allele) has been associated with lower plasma triglyceride levels and higher plasma HDL-C levels compared with those with absent X alleles, and can be considered a protective factor against the development of CAD [41].

*b*. *APOC2 Gene*

The *APOC2* gene (located on chromosome 19q13.32) encodes apolipoprotein C-II (ApoC-II) which is a cofactor needed to activate LPL, the enzyme that hydrolyzes plasma triglycerides and transfers fatty acids to tissues. Homozygous mutations in *APOC2* cause Hyperlipoproteinemia, type Ib (OMIM, 608,083), an autosomal recessive disease characterized by extremely elevated serum concentrations of triglycerides (up to 30,000 mg/dL) and chylomicrons (CM), causing recurrent pancreatitis and early atherosclerosis [16,42].

*c*. *ABCG5 and ABCG8 Genes*

Sitosterolemia (STSL) is a rare, autosomal recessive disease caused by mutations in the genes *ABCG5* (encoding sterol-1) and *ABCG8* (encoding sterol-2) located on chromosome 2p21. The presence of intestinal hyperabsorption of all sterols derivatives and the reduced ability to excrete sterols into the bile lead to elevated plasma sterol levels (>30 times the normal value), development of tendon xanthomas, accelerated atherosclerosis, and premature CAD. Most patients have homozygous or compound heterozygous mutations of the two genes involved, whereas the prevalence of heterozygous individuals and their phenotypic features are not fully known [6,43]. Mutations in the *ABCG5* gene have been reported frequently in Asian patients, whereas Caucasian patients usually have *ABCG8* mutations. However, Wang et al. [44] reported the presence of *ABCG8* gene mutations in 3 of the 8 patients from unrelated Chinese families, suggesting that *ABCG8* mutations are not present exclusively in Caucasians [44].

#### 5.1.4. Familial Combined Hyperlipidemia and Familial Hypertriglyceridemia

##### The *APOA1/C3/A4/A5* Gene Cluster and Lipid Metabolism

*a*. *Familial Combined Hyperlipidemia*

Familial Combined Hyperlipidemia (FCHL) is the most common form of primary dyslipidemia, affecting 1–2% of the Western population and 14–20% of patients with premature CAD. The manifestations of FCHL are heterogeneous, the disease may manifest itself in the form of mixed hyperlipidemia, isolated hypercholesterolemia, hypertriglyceridemia or in combination with elevated ApoB levels. Although initially considered an autosomal dominant disease with incomplete penetration, linkage analysis and GWAS have suggested that the etiology of FCHL is complex, multifactorial. The characteristic FCHL phenotype is determined by the interaction between genetic factors (several genes, of which 1 or 2 with major effect-oligogenic theory) and environmental factors. Although the etiology of FCHL is not fully elucidated, GWASs have indicated three possible loci involved: 1q21-23, 11p14.1-q12.1, and 16q22-24.1 [4].

Various studies have shown the key role of the *APOA1/C3/A4/A5* haplotype (located on chromosome 11) in modulating lipoprotein metabolism. The conclusion of the study by Liu et al. [45] was that certain variants of the *APOA1/C3/A4/A5* haplotype may be useful markers for predicting the response to fenofibrate therapy, and further confirmation is required in other studies [45]. Eichenbaum-Voline et al. [46] showed in a study that the *APOA5* c.56G>G and *APOC3* c.386G>G alleles are associated with the production of FCHL [46,47]. 

The prevalence of CAD in patients with FCHL under the age of 60 is approximately 15% [48]. Patients with FCHL have an increased risk of CVD and are frequently associated with other metabolic disorders: type 2 diabetes mellitus (T2DM), non-alcoholic fatty liver disease, steatohepatitis and metabolic syndrome. Hopkins et al. [49] identified metabolic syndrome in 65% of patients with FCHL, compared with 19% of control subjects [49].

*b*. *Familial Hypertriglyceridemia*

Familial hypertriglyceridemia (FHTG) (OMIM, 145,750) is a rare hereditary primary dyslipidemia characterized by a moderate increase in serum triglycerides (>400 mg/dL), usually in the absence of significant hypercholesterolemia [16]. FHTG has a prevalence of 5–10% in the general population and is an autosomal dominant monogenic disease that rarely manifests itself in childhood, being usually diagnosed in adulthood. Affected people are associated with obesity and decreased glucose tolerance. The metabolic cause of FHTG is the hepatic secretion of large VLDL particles rich in triglycerides that are slowly catabolized. Although the molecular defect has not yet been identified, some studies have suggested that several loci may be associated with FHTG (e.g.,15q11.2-q13.1, 8q11-q13), but further studies are needed to confirm this association [4]. The *APOA5* SNP rs2075291 (c.553G>T; p.185Gly>Cys) can be considered a susceptibility factor for hypertriglyceridemia and CAD [50]. 

The *APOA5* G553T allelic variant (which causes cysteine substitution with glycine-185) was identified by Kao et al. [51] in a study that included Chinese patients who had hypertriglyceridemia [51]. 

Do et al. [52] identified that rare *APOA5* and *LDLR* alleles increase the risk of MI; *APOA5* gene polymorphism was associated with elevated plasma TG levels, whereas carriers of *LDLR* mutations had elevated plasma LDL-C [52]. The prevalence of FHTG in families in which premature CAD occurred was analyzed in two independent studies. Hopkins et al. [50] found FHTG in 20.5% of families with at least one case of CAD; approximately 71% of patients with FHTG had metabolic syndrome, compared with 19% in the control group [50]. Genest et al. [53] identified the presence of hypertriglyceridemia in 1% and hypertriglyceridemia with hypoalphalipoproteinemia in 14.7% of families in whom there was a CAD diagnosed before the age of 60 [53].

#### 5.1.5. Atherosclerosis Susceptibility/Atherogenic Lipoprotein Phenotype

Mutations in the *ATHS* gene (located on chromosome 19p13.3-p13.2) cause Atherosclerosis Susceptibility (ATHS) also called Atherogenic Lipoprotein Phenotype (ALP) (OMIM, 108,725) [16]. ATHS/ALP is an autosomal dominant monogenic disease characterized by the presence of elevated plasma LDL levels, elevated triglyceride-rich lipoproteins levels, and decreased plasma HDL levels, and is associated with an increased risk of CAD and MI. ALP has two phenotypic variants: type A-characterized by the presence of large LDL particles and type B-characterized by the presence of small and dense LDL particles. Current data indicate an association between the presence of the type B phenotype (low, dense LDL) and an increased risk of CAD. Austin et al. [54] concluded that phenotype B may be an independent risk factor for CAD and a 3-fold higher risk of MI [54].

Nishina et al. [55] identified a link between the ALP phenotype and the *LDLR* gene locus (located on chromosome 19p13.3-p13.2) and concluded that the *ATHS* gene that causes ATHS/ALP may be the same as the *LDLR* gene or is located near the *LDLR* locus [55]. The link between the *ATHS* gene and the *LDLR* locus was later confirmed in the study by Rotter at al. [56], which suggested that the specific ALP phenotype is determined, however, by a different gene from the *LDLR* gene [56]. 

Some studies have provided important evidence for the involvement of the *CETP* gene (located on chromosome 16q13) encoding cholesteryl ester transfer protein-CETP) and the *SOD2* gene (located on chromosome 6q25.3), encoding superoxide dismutase-2-SOD2 in the production of an increased susceptibility to atherogenic dyslipidemia, as well as a possible involvement of the haplotype *APOA1/APOC3/APOA4* (located on chromosome 11p14.1-q12.1) [16,56]. Srisawasdi et al. [57] showed in a study that included 299 Thai patients treated with statins, the polymorphisms of *CETP* rs3764261 (CC genotype) and rs708272 (GG and GA genotypes) may have a higher susceptibility to atherogenic dyslipidemia [57].

Allayee et al. [58] concluded that there are common genetic factors that determine FCH, but also ATS/ALP (type B, small dense LDL particles) associated with early-onset CAD [58].

### 5.2. Other Monogenic CAD

#### 5.2.1. *MEF2A* Gene

Mutations in the *MEF2A* gene (located on chromosome 15q26.3) cause coronary artery disease, 1 (ADCAD1) (OMIM, 608,320) [16]. The *MEF2A* gene encodes a transcription factor (myocyte enhancer factor 2A) that acts in the embryonic period. The *MEF2A* gene is thought to contribute to the maintenance of vascular endothelial cell function (being involved in myocyte differentiation and vasculogenesis) and to interact with other factors involved in the pathogenesis of CVD [59].

Wang et al. [60] identified a deletion in exon 11 of the *MEF2A* gene in 10 of the 13 members affected by CAD (9 of whom had a history of MI), belonging to a large family, analyzed by genome-wide linkage analysis. The mutation was not identified in the case of family members unaffected by CAD. Deletion of seven amino acids (D7aa MEF2A) disrupts the activity of the transcription factor MEF2A associated with abnormalities in endothelial cells and vascular smooth muscle cells (VSMc) involved in the processes of atherogenesis. The results obtained led to the identification of a pathogenic mutation of a gene that intervenes in the MEF2A signaling pathway, involved in the etiopathogenesis of familial vascular disease associated with CAD/MI [60].

The involvement of the *MEF2A* gene in the pathogenesis of CAD remains controversial, as in other studies the association with familial/sporadic CAD has not been proven. Cases have been reported in which individuals with D7aa deletion *MEF2A* did not have CAD before the expected age of onset of CAD, whereas members of the same family who had CAD did not have the mutation. In some studies, the *MEF2* gene variants c.704C>A (p.S235Y), c.812C>G (p.P271R), c.836C>T (p.P279L), c.848G>A (p.G283D) missenses, c.1315C>T (p.R439X) nonsense, and seven out-of-frame deletions were predicted as disease-causing variants for CAD [59].

The allelic variants that do not alter the activity of the MEF2A transcription factor are not associated with an increased risk of CAD, and the prevalence of pathogenic *MEF2A* mutations in the general population is not yet fully known [6,61]. The phenotypic variability and penetration of the mutant gene may be due to the interaction of *MEF2A* with other modifier genes (epistasis) or environmental factors [59].

Improving or maintaining MEF2A expression in vascular endothelial cells may be a new strategy for developing vascular protection methods and exploring new vascular protective drugs. Liu et al. [62] identified a new mechanism involved in the protective role of resveratrol in promoting the expression of MEF2A in vascular endothelial cells, which in turn would influence the expression of anti-apoptosis and anti-aging genes (eg *SIRT1* gene) and thus inhibit premature apoptosis or senescence of vascular endothelial cells [62].

#### 5.2.2. *ST6GALNAC5* Gene

The *ST6GALNAC5* gene located on chromosome 1p31.1 (OMIM, 610,134) encodessialyltransferase 7e, an enzyme that modifies proteins and ceramides on the cell surface, influencing intercellular interactions and those between cells and the extracellular matrix [16]. 

InanlooRahatloo et al. [63] analyzed an consanguineous Iranian family, with cases of autosomal dominant premature CAD. GWAS combined with WES allowed the identification of a heterozygous mutation (c.G295A) in the *ST6GALNAC5* gene (which determines p.Val99Met). Targeted sequencing of all family members confirmed the co-segregation between this allelic variant and the CAD phenotype [63]. Analysis of other Iranian families with CAD identified a second heterozygous mutation p.*337Qext*20 identified in two unrelated patients. One of the patients had a brother with CAD and two unaffected siblings. Both mutations have been shown to increase sialyltransferase activity in vitro, possibly in vivo. Increased sialyltransferase activity in blood cells and serum sialic acid levels are associated with atherosclerosis and CAD [64]. Some studies in the United States have provided statistically significant additional evidence for the potential contribution of *ST6GALNAC5* gene mutations to the occurrence of CAD [63]. The evidence provided by these studies supports the idea that sialic acid and sialyltransferase activity are involved in the pathogenesis of atherosclerotic CAD, and that the gain-of-function mutations in the *ST6GALNAC5* gene are an etiological factor for CAD. The pathophysiological mechanism and the prevalence of functional mutations in the *ST6GALNAC5* gene in the general population and in patients with CAD are still unknown [63].

#### 5.2.3. *CYP27A1* Gene

Homozygous or compound heterozygous mutations in the *CYP27A1* gene (located on chromosome 2q35) cause cerebrotendinous xanthomatosis (CTX) (OMIM, 213,700), a rare autosomal recessive lipid storage disease [16]. CTX is manifested by progressive neurological dysfunction (cerebellar ataxia with postpubertal onset, systemic damage to the spinal cord and a pseudobulbar phase leading to death), premature atherosclerosis and cataracts [16].

In the study by Inanloo Rahatloo et al. [65], the analysis of CAD patients using WES led to the identification of the *CYP27A1* c.G674A mutation that causes p.Arg225His protein substitution. The mutation of the *CYP27A1* gene affects the function of the enzyme sterol 27-hydroxylase, which is involved in the transport and elimination of cholesterol from cells, and this mechanism can be correlated with CAD phenotype [65]. In another study, the analysis of a group of 100 unrelated CAD patients using WES identified the presence in seven of them of four different *CYP27A1* allelic variants (p.Arg14Gly, p.Arg26Lys, p.Ala27Arg and p.Val86Met) that could cause CAD [65]. Chen at al. [66] identified three new mutations in *CYP27A1* (p.Arg513Cys, c.1477-2A>C and p.Arg188Stop (NM 000784.3) in a study that included four Chinese families with CTX [66]. Lee at al. [67] reported another pathogenic mutation in the *CYP27A1* gene present in two Taiwanese brothers with CTX [67]. Both brothers had a compound heterozygous genotype with a mutation in exon 2 (c.435G>T, cryptic splice site) and a mutation in intron 7 (c.1264A>G, canonical spice site) [67]. In another study that analyzed members of an Iranian family affected by CTX, Rashvand et al. [68] identified a homozygous splicing mutation, NM_000784: exon6: c.1184+1G>A in the *CYP27A1* gene, that was present in most cases [68]. The results of these studies confirm that mutations in the *CYP27A1* gene, which regulate cholesterol homeostasis, can lead to atherosclerosis [66,67].

#### 5.2.4. *LRP6* Gene

Mutations in the *LRP6* gene (located on chromosome 12p13.2) encoding low-density lipoprotein receptor-related protein 6 (LRP6) cause Coronary artery disease, autosomal dominant, 2 (ADCAD2) (OMIM, 610,947) [16]. ADCAD2 is characterized by an increased risk of MI in the presence of increased metabolic risk factors.

Mani et al. [69] analyzed a family with autosomal dominant premature CAD, in which family members had clinical manifestations specific to the metabolic syndrome (hyperlipidemia, hypertension, DM) and osteoporosis [69]. In these patients, they identified a R611C homozygous missense mutation (which substitutes cysteine for arginine) in the *LRP6* gene, which encodes a co-receptor in the Wnt signaling pathway [69]. These could be an important evidence of the influence of Wnt signaling pathway abnormalities on cardiovascular risk factors (CRF). Most heterozygous individuals over the age of 45 had clinical manifestations of metabolic syndrome, suggesting that the impact of *LRP6* mutation on mulftiple CAD risk factors is important, and the ubiquitous expression of *LRP6* gene could explain pleiotropic manifestations in various tissues [69]. 

Loss-of-function mutations in the *LPR5* and *LPR6* genes cause decreased bone density and osteoporosis. 

In addition, recent studies have shown a strong association between osteoporosis and CAD, which may be the pleiotropic effects of mutations in the Wnt signaling pathway. Intronic mutations of *TCFL* gene and other transcription factors involved in the Wnt signaling pathway are associated with type 2 diabetes mellitus (T2DM) and maturity-onset diabetes of the young (MODY diabetes). In the future, investigating the factors that interfere with the Wnt signaling pathway in patients with premature CAD and metabolic syndrome could provide new perspectives into the pathophysiology of the disease and the use of more effective prophylactic measures [69].

Wang et al. [70] analyzed 766 Chinese patients with CAD and concluded that the *LRP6* gene polymorphism (the C allele of the SNP rs11054731 located in intron 2) is associated with increased susceptibility and severity of CAD [70].

## 6. Polygenic CAD: Genes and Polymorphisms Associated with CAD

Monogenic mutations explain only a small part of the etiology of CAD/MI, being recognized that the etiology of CAD is complex and the variable phenotype results from the interaction between many genes (polygeny) and environmental factors. The large number of genes, possibly involved, and the insufficient knowledge of the pathophysiological mechanisms through which they intervene in the production of the disease have created difficulties in the attempts to elucidate the etiology of CAD over time. The development of molecular technologies in the last two decades has allowed extensive analysis of the whole genome (GWAS) or exome (WES), which provided important information on the role of genetic factors in the etiology of multifactorial diseases (heritability).

*Candidate gene-based association studies* (CGS) have made little contribution to elucidating the genetic etiology of CAD or other multifactorial diseases. The cause could be the low reproducibility correlated with the small number of cases analyzed, which lead to results with low statistical power for the identification of less associated allelic variants [10].

*Genome-wide association studies* (GWAS) examine the co-segregation of polymorphic genetic markers (SNPs-single nucleotide polymorphism) distributed throughout the genome in families affected by CAD. A rare allelic variant present in 1% of the population is considered polymorphism. It is estimated that approximately 3,000,000 SNPs (one SNP in every 1000 base pairs) are present throughout the genome (3 billion base pairs) [10].

Early GWASs showed a reduced association of common allelic variants with CAD with odds ratio (OR) ranging from 1.1 to 1.4. The need to identify these allelic variants and to confirm the results through other independent studies has extended international collaborations, which has allowed the analysis of a very large number of samples [10].

The first two GWASs (2007) identified an association of SNPs located in the 9p21 chromosomal region with CAD and MI, data that were later confirmed by other studies that included large cohorts of patients and a broad ethnic, geographical and demographic spectrum [10,71,72]. Many studies have subsequently confirmed the association with CAD and MI of the 9p21.3 chromosomal region, which contains two cyclin-dependent kinase inhibitors, *CDKN2A* (encoding the prototypic INK4 protein, p16INK4a) and *CDKN2B* (encoding p15INK4b), which are linked to both DM as well as the pathogenesis of atherosclerosis by their role in inhibiting TGF-β-induced cell growth [10,73].

Nikpai et al. [74] performed the largest GWAS meta-analysis that included 185,000 CAD cases and controls, in which they analyzed 6.7 million common (minor allele frequency, MAF > 0.05) and 2.7 million low-frequency (0.005 < MAF < 0.05) variants [74]. In addition to the loci already known to be associated with CAD, they identified 10 new loci containing candidate genes involved in the processes occuring in the vascular wall [74]. They observed the presence of allelic heterogeneity, providing evidence that genetic susceptibility to CAD is determined by common allelic variants (SNPs), without providing evidence of the association between rare alleles and CAD [74].

Another GWAS conducted by the CARDIoGRAMplusC4D Consortium which included a total of 63,746 CAD cases and 130,681 controls identified/confirmed another 15 susceptibility loci for CAD. At that time, the total number of loci known to be associated with CAD was 46. In addition, another 104 independent variants (r^2^ < 0.2) strongly associated with CAD were identified [6,75]. Together, these variants explain 10.6% of CAD heritability. In total, 12 of the 46 genome-wide significant SNPs showed a significant association with lipid metabolism, and 5 were significantly associated with blood pressure, but none showed a significant association with DM [6,75]. A GWAS by Wang et al. [76] in the Chinese Han population indicated that the SNP variant (rs6903956) in the *C6orf105* gene (located on chromosome 6p24.1) is associated with susceptibility to CAD [76].

In the study published by IBC 50K CAD Consortium, other new susceptibility loci for CAD were reported: *COL4A1/COL4A2*, *ZC3HC1*, *CYP17A1* [77]. In a recent large-scale GWAS, Koyama et al. [78] identified 43 new loci associated with increased CAD susceptibility in ethnic Japanese people, not previously reported in other studies [78]. Matsunaga et al. [79] identified three new loci associated with CAD located on chromosomes 1q21 (*CTSS*), 10q26 (*WDR11-FGFR2*) and 11q22 (*RDX-FDX1*) highlighting the genetic differences of ethnic Japanese people compared with the European population [79].

In a German study that combined the results of the Wellcome Trust Case Control Consortium (WTCCC) study and the German MI [Myocardial Infarction] Family Study, along with the 9p21.3 locus, two loci were strongly associated with CAD (adjusted *p* < 0.05), being located on chromosomes 6q25.1 (rs6922269) and chromosome 2q36.3 (rs2943634). Combining the results of the two studies, four additional loci were identified that are associated with a high probability (over 80%) with CAD (*p* < 1.3 × 10^−6^), located on chromosomes 1p13.3 (rs599839), 1q41 (rs17465637), 10q11.21 (rs501120) and 15q22.33 (rs17228212) [73]. 

In the chromosomal region 1p13.3 is located the *PSRC1* gene, which encodes a proline-rich protein, and in the chromosomal region 1q41 is the locus of the *MIA3* gene (melanoma 3 inhibitory activity gene), also called *RNA* or *TANGO* [73,80]. Polymorphisms (SNPs) associated with the 10q11.21 chromosomal region are located 100 kb downstream of the *CXCL12* (the stromal cell-derived precursor factor 1) gene. The SNP on chromosome 15q22.33 is an intronic SNP in the *SMAD3* gene, which functions as a transcriptional regulator activated by transforming growth factor β (TGF-β) and activin receptor-like kinase 1 (ALK1) [73,81,82,83].

Although initially 55 loci associated with CAD/MI were identified, subsequently, starting with 2007, numerous studies have led to the identification of over 321 loci of susceptibility to CAD and MI [78,80,81,82,83,84]. The most common allelic polymorphisms identified by recent GWASs in patients with CAD/MI are shown in Table 2.

**Figure 2:**
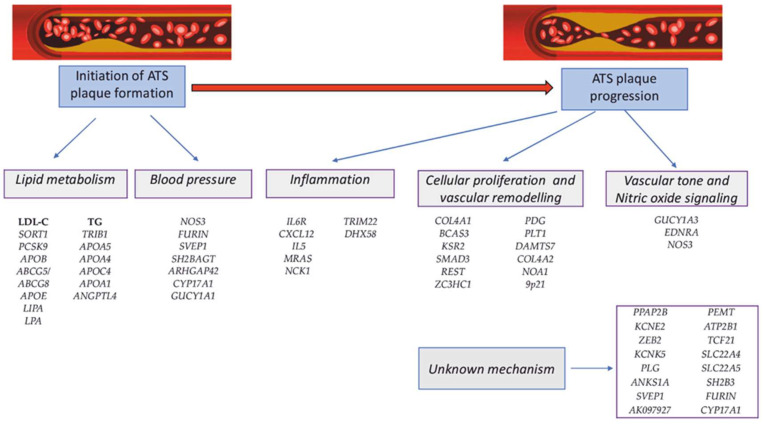
The main genes involved in the pathophysiological mechanism of CAD. CAD: Coronary artery disease; LDL-C: Low-density lipoprotein cholesterol; TG: Triglyceride; ATS plaque: Atherosclerotic plaque.

The data obtained were extremely important, because the implementation of polygenic risk scores (PRS) in clinical practice largely depends on the accuracy of predicting the magnitude of the effect of risk alleles, which vary depending on the genetic background [80]. Together, these allelic variants explain approximately 15% of CAD/IHD heritability. Most CAD-associated polymorphisms were from genes involved in lipid metabolism, blood pressure regulation, and inflammation, confirming the importance of these factors in the etiology of CAD. In addition, many of the CAD-associated genes are involved in the metabolism of amino acids, polyamines, innate immunity, and degradation of the extracellular matrix (Figure 2) [10,84].

Most of these mutations are located in intergenic regions, at or near the promoters, indicating a possible influence on gene expression by epigenetic regulation, as a possible mechanism of CAD/IHD [85].

The consistent results, sometimes achieved through extensive collaborations between different study groups, as well as the availability of data obtained from the scientific community are the main benefits of GWASs. Currently available GWAS databases include IHD-associated genetic variants from the CARDIoGRAMplusC4D consortium, European Genome-phenome Archive and American database of Genotypes and Phenotypes (dbGaP) [10,75]. Identifying the still unknown genetic factors involved in the etiology of complex, multifactorial diseases is one of the major challenges in the case of CAD. Heritability, still unknown, may be due to unidentified variants of the disease associated genes or may be due to factors that do not alter gene structure but wich influence the intensity of gene expression by epigenetic regulation [6]. 

The major limitations of GWAS are related to the fact that the identified allelic variants could be in a Linkage disequilibrium (LD) with the disease-causing alleles and do not provide any information about the associated pathophysiological mechanism, which must be subsequently identified by specific functional studies. In addition, the definition of the clinical phenotype being studied can vary in different study groups. GWAS sensitivity is limited to high frequency variants (5%). GWASs aim is to identify common allelic variants, which have small, additive effects, being less effective in identifying rare allelic variants with major effects. GWASs (SNPs) have low power to identify structural abnormalities (CNVs caused by deletions, insertions, translocations), as well as a low power to identify both interactions between genes, and the interaction between genetic and environmental factors. To overcome these limits, different strategies have been proposed: the correct definition of the phenotype of the analyzed cases, the increase in the number of patients included in the analyzed groups and the use of groups with extreme phenotypes; development of powerful biostatistical tools to increase the sensitivity of the detection rate, especially of rare allelic variants; fine mapping of SNPs or the use of NGS (next-generation sequencing) for regions of interest to identify rare allelic variants and/or structural variants; taking into account the mechanisms of epigenetic regulation of gene expression [6,10].

### 6.1. Polymorphism of Genes Involved in Lipid Metabolism: A Novel View

#### 6.1.1. Polymorphisms of *APOE* Gene and CAD

Apolipoprotein E (ApoE) is an LDL receptor (LDLR) ligand and is encoded by the *APOE* gene located on chromosome 19q13.32 that is closely related to the *APOC-I/C-II* gene complex. Through LDLR, ApoE is involved in the elimination of very low density lipoprotein (VLDL) residues and chylomicrons (CM) [84,85,86]. There are three *APOE* allelic variants (ε2, ε3 and ε4) in the European population, which determine six genotypes (*APOE2/2*, *APOE2/3*, *APOE2/4*, *APOE3/3*, *APOE3/4*, *APOE4/4*), which encode three major ApoE isoforms (ApoE2, ApoE3 and ApoE4) [86,87]. Their presence correlates with a variable affinity for LDLR, resulting in significant differences in plasma total cholesterol (CT) and LDL-C levels. Many studies have shown that *APOE* polymorphism is associated with CAD and increased risk of MI. The frequency of the *APOE*ε4 allele in the European population is about 15% and is associated with early atherosclerosis, increased mortality, risk of ischemic stroke, Alzheimer’s disease and MI [86,87]. Heterozygous carriers of the *APOE*ε4 have an 8.3% higher LDL-C levels than individuals *APOE*ε3 homozygotes. Heterozygous carriers of the *APOE*ε2 allele have 14.2% lower plasma LDL-C levels compared with *APOE*ε3 homozygotes [86,87]. Various studies have shown that individual carriers of the *APOE*ε4 allele have a 40% higher risk of death from CAD than people with the *APOE3*/3 genotype or *APOE*ε2 carriers [88].

In the study by Gerdes et al. [89], men carrying *APOE*ε4 allele had a 1.8-fold increased risk of death from CAD compared with carriers of other *APOE* alleles [89]. 

Humphries et al. [90] found that the relative risk of developing CAD is dependent on the interaction between *APOE* genotype and smoking status, suggesting that the interaction between genetic and environmental factors is involved in the variable expressivity of CAD related to *APOE* genotype [90]. These results were not confirmed by the Whitehall II study, which, although confirming the protective effect of the *APOE*ε2 allele, did not reveal a higher risk for CAD in non-smokers carrying the *APOE*ε4 allele, all smokers having a similar risk for CAD, regardless of the *APOE* genotype [91].

#### 6.1.2. Polymorphisms of *APOB* Gene and CAD

The *APOB* gene mutations cause Familial Defective Apolipoprotein B-100 (FDB), an autosomal dominant disease associated with an increased risk of atherosclerosis and CAD [6,21]. Over time, there have been studies that have examined the association between *APOB* polymorphisms and the increased risk of CAD in individuals undiagnosed with FDB. The *APOB* gene has numerous polymorphic loci, three of which (XbaI, EcoRI, SpIns Del) are correlated with elevated plasma total cholesterol (TC), LDL-C, ApoB and triglyceride (TG) levels and an increased risk of developing CAD/MI.

The results obtained by Chiodini et al. [92] demonstrated that *ApoB Eco*RI (although rare) and *SpIns/Del* polymorphisms significantly increase the risk of CAD and MI, but these results need further confirmation by other studies [92]. 

The *X1*, *R1*, and *ID1* polymorphisms were more common in patients with a history of MI than in the control group, in a study by Hegele et al. [93]; there were no significant differences in plasma LDL-C or ApoB levels between the two groups [93]. A meta-analysis by Chen et al. [94] indicated that *ApoB Eco*RI polymorphism is associated with a moderate risk for CAD, and the E^−^ allele at this locus could be a susceptibility allele for CAD development [94].

#### 6.1.3. Polymorphisms of *LPL* Gene and Its Modulators Associated with High Risk of CAD

Different types of homozygous or heterozygous compound mutations in the *LPL* gene cause Lipoprotein lipase deficiency (LPLD deficiency), a rare autosomal recessive monogenic disorder. Lipoprotein lipase (LPL) is an enzyme involved in the metabolism of triglycerides-rich lipoproteins and acts on the vascular level. Over time, there have been many studies that have reported an association between *LPL* polymorphisms, and CAD/MI, in some cases the results being contradictory. The *LPL* locus can alternatively be occupied by both common, non-coding and rare allelic variants. Rare loss-of-function mutations in *LPL* gene are associated with an increased risk of CAD, and gain-of-function mutations in *LPL* gene are associated with reduced risk of CAD [80].

Several meta-analyses suggested that, compared with non-carriers, heterozygous individuals with Gly188Glu, Asp9Asn and Asn291Ser substitutions have an atherogenic lipoprotein profile, whereas carriers of Ser447Ter substitution have a protective lipoprotein profile [80].

In a recent meta-analysis, He et al. [95] showed that *LPL* HindIII and S447X polymorphisms but not PvuII could act as protective factors against MI, requiring further confirmation by other case–control studies with a larger number of subjects analyzed [95]. Ma et al. [96] did not identify in their meta-analysis any significant association for *LPL* N291S and PvuII polymorphisms and CAD. Analyzing the results according to ethnicity, they observed a significant correlation between *LPL* S447X polymorphism and CAD susceptibility in Caucasians, an autosomal dominant transmitted variant. The *LPL* D9N polymorphism was associated with an increased risk of CAD, whereas S447X and HindIII polymorphisms showed protective effects. No association was observed between *LPL* N291S and PvuII polymorphisms and risk of CAD [96]. Talmud et al. [97] analyzed 2708 healthy middle-aged European men and found that smokers with *LPL* Asp9Asn polymorphism had a 10.4-fold higher risk of IHD/CAD compared with non-smoker individuals who do not carry that mutation; individuals who smoked but did not carry the allele had a 1.6 times higher risk than non-smokers [97]. No association was identified between the *LPL* Asn291Ser allelic variant and the increased risk of IHD/CAD [97]. Along with the *LPL* gene, the predisposition to CAD is also determined by the genes that regulate its endogenous activity, such as the *APOA5, APOC3* and *ANGPTL3* genes [80,98].

#### 6.1.4. Polymorphism of *OLR1 (LOX1)* Gene and CAD

The *OLR1* (*LOX1*) (oxidized low-density lipoprotein receptor 1) gene is located on chromosome 12p13.2 and encodes a low-density lipoprotein receptor (LDLR) that belongs to the C-type lectin superfamily. The LDLR protein binds, internalizes, and degrades oxidized LDL, and may be involved in the regulation of apoptosis. Gene regulation is mediated by the cAMP signaling pathway. The *OLR1* gene mutations have been associated with atherosclerosis and an increased risk of CAD/MI and Alzheimer’s disease in various studies [16].

Earlier studies indicated the association of *OLR1* K167N polymorphism and CAD/MI, as well as a different frequency of the homozygous genotype IVS4–73T/T in individuals with MI and in the control group without MI. These particularities were contradicted by the study of Trabetti et al. [99], which considers that they are correlated with genetic differences between different ethnic groups, as well as with the limited number of patients analyzed [99]. 

SNPs located in the *OLR1* gene could have clinical significance and could be considered CAD candidate biomarkers, their identification being useful in assessing the genetic risk of CAD. The *OLR1* gene has six non-coding SNPs, which form a haplotype [100]. In a meta-analysis, Salehipour et al. [100] identified a significant association between SNPs rs1050283 (3′UTR*188 C>T) and rs3736235 (IVS4-14 A>G) located in *OLR1* haplotype and the occurrence of CAD [100]. They suggested that the precise determination of CAD association with polymorphisms located in a haplotype requires the analysis of all SNPs located in that specific haplotype [100].

#### 6.1.5. Other Genetic Polymorphisms Involved in Lipid Metabolism Associated with Increased Risk of CAD

*a*. *SORT1 Gene*

The *SORT1* gene (sortilin 1), located on chromosome 1p13.3, identified by the first GWASs, encodes sortilin 1, which plays an important role in regulating plasma LDL-C levels by interacting with *APOB* gene in the Golgi apparatus in hepatocytes. Several studies have reported an association between plasma sortilin levels and cardiovascular damage and DM, which led to the idea that circulating sortilin plasma levels should be used as a potential biomarker for cardiovascular disease (CVD) and DM [101]. However, the results obtained in various studies are not conclusive, possibly in correlation with the small number of patients included in the analyzed samples [101]. Møller et al. [101] genotyping and sequencing the entire genome and corroborating the data obtained with plasma sortiline levels in 1173 patients with stable angina pectoris (diagnosed by computer angiography) [101]. 

Thus, two independent *cis* protein quantitative trait loci (pQTL) on chromosome 1p13.3 were identified, one of which is already known to be associated with CAD. In contrast, there was no association between circulating sortilin levels and coronary artery calcium score (CACS) or disease severity [101]. They concluded that although low sortilin levels are associated with the risk of CAD, its effect size is too small for sortilin to be a useful biomarker for CAD in medium or low-risk chest pain patients [101].

*b*. *TRIB1 Gene*

The *TRIB1* gene located on chromosome 8q24.13 encodes pseudokinase 1 involved in hepatic lipid metabolism by influencing the expression of lipogenic genes, and is also associated with insulin resistance. However, its mechanisms of action are not fully elucidated. The *TRIB1* locus has been linked to the metabolism of hepatic triglycerides in mice and to plasma triglyceride levels and CAD in humans [16]. GWASs indicated a SNP located at ≈30 kb downstream of *TRIB1* gene, which would have complex regulatory effects on genes or pathways involved in hepatic triglyceride (TG) metabolism. Some studies suggest that *TRIB1* gene suppresses the transcriptional activity of the *FOXO1* gene, thus suppressing the expression of the G6Pase and Phosphoenolpyruvate carboxykinase (PEPCK) enzymes, limiting gluconeogenesis [80,102]. Douvris et al. [103] identified by GWAS the *TRIBAL* locus that interacts with the *TRIB1* locus. The *TRIBAL* locus has a risk SNP that influences the expression of the *TRIB1* gene and is associated with elevated plasma TG levels [103].

### 6.2. Polymorphism of Genes Involved in Vascular Homeostasis

The formation and growth of atheroma plaque in the coronary wall is a slow and progressive process in which the endothelium is a key player. The number of risk genes which can generate vascular wall dysfunction has increased with deciphering the complex pathophysiological mechanisms of atherosclerosis, including the role of innate immunity and prothrombotic factors. Through synergistic action, they lead to vascular obstruction and cardiomyocyte ischemia [4,6].

#### 6.2.1. Genes Involved in the Function of Vascular Smooth Muscle Cells (VSMc)

Vascular smooth muscle cells (VSMc) can be involved in the formation of atherosclerotic plaque by at least two mechanisms: modulating blood pressure through vascular tone and vascular remodeling, which, together with other factors, lead to either stabilization or progression and rupture of the atherosclerotic plaque [4,6].

*a*. *Endothelial Cell Nitric Oxide Synthase 3 (NOS3) Gene Polymorphism*

The *NOS3* gene located on chromosome 7q36.1 encodes the nitric oxide endothelial cell synthase (eNOS), an enzymatic protein with 1203 amino acids and 133 kDa, expressed in vascular endothelial cells, cardiomyocytes and platelets. eNOS causes the release of nitric oxide (NO) at the vascular level which causes the relaxation of vascular smooth cells. NO also has the physiological role of preventing the formation of atherosclerotic plaques by inhibiting the proliferation of smooth muscles and the adhesion and aggregation of platelets. Various studies have suggested that changes in vascular NO levels disrupt vascular homeostasis, causing endothelial dysfunction and may play a role in the etiopathogenesis of CAD [104]. Studies in mice have shown that the absence of nitric oxide receptor in vascular smooth muscle cells (VSMc) causes hypertension [4,104].

*NOS3* gene polymorphism may influence eNOS enzyme synthesis. *NOS3* expression is regulated by epigenetic mechanisms (DNA methylation) and micro-RNA molecules. The localisation and activity of the eNOS protein is regulated by post-translational mechanisms (phosphorylation or acetylation).

Li at al. [105] analyzed the association between genetic variants of the *NOS3* gene and the risk of CAD in a meta-analysis that included 132 GWASs [105]. Of the thirteen *NOS3* allelic variants analyzed, the polymorphisms rs891512, rs1799983, rs2070744, rs11771443 and rs869109213 had a significant association with CAD and could serve as genetic biomarkers of CAD [105]. Three of these (rs1799983, rs2070744 and rs869109213 polymorphisms) were significantly correlated with the risk of MI and ACS. The rs869109213 polymorphism was common in Caucasians, and rs1799983 and rs2070744 polymorphisms were significant in both Caucasians and the Asians [105]. 

Gholami et al. [106] analyzed the study of Lin et al. [105] and completed the results obtained by them, concluding that, in the case of the five polymorphisms significantly associated with CAD, major alleles of rs1799983 (G), rs2070744 (T) and rs869109213 (4b) showed protective effect for CAD [105,106].

*b*. *TCF21 Gene Polymorphysm*

The *TCF21* gene (located on chromosome 6q23.2) encodes a transcription factor (TCF21) expressed in the epicardium during the embryonic period, which plays a role in differentiating epicardial cells. Recently, data on the role of TCF21 in the etiology of atherosclerotic CAD have been published. The loss-of-function mutation of *TCF21* gene during the embryonic period would be associated with premature differentiation of coronary vascular smooth muscle cells (VSMc) into the pericardium, leading to decreased migration of VSMc into the myocardium. The association of *TCF21* polymorphism with CAD has been shown in populations of different ethnicities [80,107].

Wirka et al. [107] showed that *TCF21* deficiency inhibited the VSMc phenotypic switch to fibromyocytes which reduces the number of fibromyocytes in the fibrous cap of atherosclerotic plaques, which thus become unstable [107]. In addition, downstream of the *TCF21* gene there are known susceptibility loci for CAD suggesting that *TCF21* gene could play a major role in the atherosclerotic plaques formation by epigenetic regulation of the expression of other genes [80,108]. A pathogenic mutation in the 3 ‘UTR region of the *TCF21* gene impairs mRNA stability by differentiated binding of a microRNA. Directing the interaction between microRNA and mRNA by oligomers could be an effective therapeutic target for coronary VSMc [80].

*c*. *ADAMTS7 Gene Polymorphism*

The *ADAMTS7* gene located on chromosome 15q25.1 encodes a zinc-dependent protease expressed in the extracellular matrix. ADAMTS7 is a transmembrane protein that is involved in both the interaction between proteins and in multiple processes in the body, including signaling, adhesion, and cell migration. ADAMTS7 is synthesized in vascular endothelial cells and VSMc and has been shown to degrade several members of the thrombospondin family. Currently, the mechanisms by which ADAMTS7 determines CAD are not fully elucidated. ADAMTS7 protein promotes VSMc migration and neointima formation after injury by degradation of a cartilage oligomeric matrix protein [80]. Mice deficient in Adamts7 develop less atherosclerosis and are resistant to the formation of neointima secondary to vascular damage [80]. Three different GWASs identified the 15.25.1 locus and the *ADAMTS7* gene as being associated with coronary atherosclerosis. The main SNP associated with CAD was rs3825807, which determines the substitution of adenine for guanine, resulting in a serine-proline substitution in the ADAMTS7 prodomene, which would affect the maturation of ADAMTS7 protein. None of the studies reported an association between *ADAMTS7* gene polymorphism and increased mortality in CAD patients [109].

*d*. *HHIPL1 Gene Polymorphism*

GWASs have identified a possible new CAD-associated locus on chromosome 14q32, occupied by the *HHIPL1* gene (*hedgehog interacting protein-like 1*) which encodes a homologous sequence of an antagonist of the hedgehog signaling pathway. The function of *HHIPL1* gene and its role in atherosclerosis is not fully understood. The *HHIPL1* gene is involved in the development of coronary vascularization in the embryonic period. HHIPL1 is a proaterogenic protein that enhances hedgehog signaling and regulates VSMc proliferation and migration. In experimental animal models, HHIPL1 deficiency attenuates the development of atherosclerosis by reducing VSMc proliferation and migration. Inhibition of HHIPL1 protein function could provide a new therapeutic strategy for CAD [110].

#### 6.2.2. Genes Involved in Blood Pressure Regulation

*a*. *Angiotensin Converting Enzyme (ACE), Angiotensin II Type I Receptor (AGTR1) and Angiotensinogen (AGT) Genes Polymorphism*

The *ACE* gene (located on chromosome 17q23.3) encodes the angiotensin converting enzyme (ACE) that converts angiotensin I to angiotensin II. The effects of angiotensin II are mediated by the angiotensin II type 1 receptor (AGT1R), encoded by the *AGTR1* gene (located on chromosome 3q23). Angiotensinogen (AGT) (encoded by the *AGT* gene located on chromosome 1q42.2) and ACE play an important role in regulating blood pressure, whereas AGTR1 plays a major role in the etiology of many cardiovascular diseases (CVD). Numerous studies have been conducted over time that have analyzed the involvement of the renin-angiotensin system and its components in the development of CAD and MI [80]. 

The *ACE* I/D polymorphism (rs4646994) is characterized by the presence (I) or absence (D) of a 287 bp Alu repeat sequence in intron 16, resulting in 3 genotypes-DD, II and ID. It is suspected that the presence of the homozygous DD genotype would be associated with the appearance of a severe form of CAD, whereas the homozygous II genotype could have a protective effect on CAD development. In their study, Amara et al. [111] showed that individuals who smoked, homozygous DD and heterozygous I/D had an increased risk of CAD, confirming the results of previous studies [111]. The association of the DD genotype with the increased risk of MI has been supported by some smaller studies, but has not been confirmed by other studies that have included a large number of patients [80]. 

In a large case–control study, Lindpainter et al. [112] did not identify an association between the presence of the D allele and an increased risk of IHD/CAD or MI [112]. Additionally, in a multicenter case–control study Keavney et al. [113] did not identify an association between the *ACE* DD genotype and an increased risk of MI [113]. On the other hand, Borai et al. [114] identified that the concomitant presence of *ACE* (I/D) and *AGT* (M235T) polymorphisms increases the risk of developing CAD, and each of the *ACE* D and *AGT* T alleles could be considered an independent risk factor for CAD [114]. 

Many studies have analyzed a possible association between the *AGTR1* gene A1166C polymorphism and CAD, but the results have been controversial. A meta-analysis by Feng et al. [115] showed that the polymorphism of the *AT1R* gene A1166C was associated with the risk of MI, with a significant association between the C allele and susceptibility to MI, whereas the AA genotype played a protective role [115]. The *AGTR1* gene A1166C polymorphism has been associated with an increased risk of hypertension in Asian and Caucasian populations, but not in Africans [116].

*b*. *CYP11B2 Gene Polymorphism*

The *CYP11B2* gene (located on chromosome 8q24.3) encodes aldosterone synthetase (ALDOS) and is regulated by angiotensin II. Several recent studies have shown that the *CYP11B2* gene rs1799998 -344C/T polymorphism is correlated with the presence of cardiovascular disease (CVD) [117,118]. A meta-analysis by Wang et al. [118] showed that the allelic variant 344CC is a risk factor for CAD and ischemic stroke in the Chinese population [118]. The presence of the *CYP11B2* -344C allele was associated with an increased risk of MI in smokers and those with dyslipidemia in a Finnish study [119].

### 6.3. Genes Associated with Vascular Hemostasis: Role of Hemostatic Gene Polymorphisms in CAD

The arterial thrombi formation is a complex and dynamic pathological process that is initiated in a damaged atherosclerotic plaque, which can completely block blood flow, producing MI. The process involves many factors, including collagen plaque components, platelet collagen receptor (glycoprotein Ia/IIa and glycoprotein VI), and blood clotting proteins [4,9].

#### 6.3.1. *ITGA2* Gene

The platelet glycoprotein (GP) complex Ia/IIa (GPIa/IIa-integrin alpha-2/integrin beta-2) (especially GPIa) is a major collagen receptor and plays an important role in platelet adhesion and aggregation with the initiation of vascular thrombosis. The phenotypic variability of this complex is closely related to the polymorphism of the *ITGA2* gene (located on chromosome 5q11.2) which encodes GPIa (integrin alfa 2). There are different opinions about the importance of the *ITGA2* gene C807T polymorphism which is thought to be associated with the risk of MI or ischemic stroke. Along with the C807T polymorphism associated with variable expression of the GPIa/IIa receptor, another *ITGA2* A1648G polymorphism was associated with changes in the tertiary structure of GPIa [4]. Kroll et al. [120] analyzed a group of 2163 Caucasian men with CAD (diagnosed by coronary angiography), showing that the *ITGA2* gene A1648G polymorphism plays an important role in the development of CAD [120].

Santoso et al. [121] analyzed a group of 2237 men with CAD (diagnosed by angiography), in which they investigated the association between *GPIa* (C807 and T807 alleles) polymorphisms and CAD/MI. No significant association with CAD/MI was observed in the total sample for any of these variants. However, the T807 allele was strongly associated with non-fatal MI in homozygous or heterozygous patients under the age of 62, with the highest risk in young heterozygous patients under the age of 49 [121].

#### 6.3.2. *Glycoprotein IIb/IIIa Platelet Receptor* Genes (*ITGB2* and *ITGB3* Gene Polymorphisms)

Fibrinogen is the major ligand of the platelet receptor GPIIb/IIIa. The polymorphism of the *ITGB2* gene (located on chromosome 21q22.3) encoding glycoprotein IIb (GPIIb-beta-2 integrin) and the *ITGB3* gene (located on chromosome 17q21.32) encoding glycoprotein IIIa (GPIIIa-integrin beta-3) were analyzed, in connection with a possible causal association in patients with CAD/MI [16,122].

The study by Reiner et al. [122] included a group of 68 young women (aged between 18 and 44 years) who have had an MI, in which they analyzed the association with GPIIb polymorphism. The study also included a control group (369 unaffected women under the age of 44) [122]. Women homozygous (Ser843/Ser843) or heterozygous (Ser843/Ile843) for the allelic variant Ser843 of the *ITGB2* gene had a significant risk of MI (1.85 times higher) compared with the control group. The risk was higher among young women who had a positive family history, who smoked, or who had hypercholesterolemia [122]. The *ITGB2* Ser843 allelic variant did not have a significant association with MI in the Japanese male population [123]. 

The *PlA1/PlA2* (*HPA1-a/HPA-1b*) polymorphisms of the *ITGB3* gene encoding GPIIIa are associated with altered beta-3 subunit conformation and increased fibrinogen binding [124]. 

In a meta-analysis that included 57 studies eligible for statistical analysis (which included 17,911 patients and 24,584 controls), Floyd et al. [124] showed that individuals carrying the *PlA2* allele (*PlA1*/*PlA2* heterozygous genotype) had a significantly increased risk of MI (*n* = 40,692; OR 1.077, 95% CI 1.024–1.132; *p* = 0.004). The degree of association with MI increased with decreasing age of subjects (≤45 years: *n* = 9547; OR 1.205, 95% CI 1.067–1.360; *p* = 0.003) and with adjustment of data for conventional cardiovascular risk factors (CRF) (*n* = 12,001; OR 1.240, 95% CI 1.117–1.376; *p* < 0.001). The study concluded that in young patients, the relative absence of conventional cardiovascular risk factors (CRF) results in a significant association between the presence of the *PlA2* allele and the risk of MI. The relationship between *PlA1/PlA2* polymorphism and MI still needs further studies [124]. In a Finnish study, young men (<40 years old) with *PlA2/PlA2* homozygous genotype had a 3–4-fold increased risk of IHD and MI [125].

#### 6.3.3. *Plasminogen Activator Inhibitor 1* (*PAI-1*) Gene Polymorphism

The *PAI-1* gene (located on chromosome 7q22.1) encodes the plasminogen activator inhibitor 1 (PAI-1) which plays an important role in regulating thrombosis and intravascular thrombolysis. Many studies have analyzed the association between the 4G/5G polymorphism of *PAI-1* gene promoter and CAD. A common *PAI-1* 4G allele is associated with elevated levels of circulating PAI-1 and allows activator transcription factor binding to the promoter, without binding to transcription inhibitory factors, such as in the case of the 5G allele [126].

In the meta-analysis by Liang et al. [126], which analyzed the results of 53 studies, it is argued that *PAI-1* 4G/5G polymorphism may contribute to individual susceptibility to CAD, but to further evaluate gene-gene and gene-environment interactions on *PAI-1* gene 4G/5G polymorphism and CAD, more studies are needed in selected populations, from different environmental backgrounds or where different risk factors are present [126]. The *PAI-1* 4G/4G polymorphism causes deficient fibrinolytic activity, and may be a useful marker of fibrinolytic activity, increasing the risk of CAD [127].

#### 6.3.4. *Thrombospondin (TBHS)* Genes Polymorphisms

The thrombospondin family (TSPs) includes 5 multifunctional glycoproteins (subgroup A: TSP-1, TSP-2 and, subgroup B: TSP-3, TSP-4, TSP-5), secreted in the extracellular matrix, with antiangiogenic functions. Zhang et al. [128] performed a meta-analysis that included 13 studies (10,801 cases and 9381 controls) in which they analyzed the association between SNP polymorphism in genes encoding thrombospondin-1 (*THBS1*-located on chromosome 15q14), thrombospondin-2 (*THBS2*-located on chromosome 6q27 and thrombospondin-4 (*THBS4*-located on chromosome 5q14.1) and the risk of CAD. The conclusion of the study was that *THBS1* N700S polymorphism was associated with an increased risk of CAD especially in the European and Asian population, whereas the *THBS4* A387P allelic variant had a significant association with CAD in the American population; no association was observed between *THBS2* 3 ‘UTR polymorphism and CAD risk [128].

#### 6.3.5. *Factor V Leiden* (*F5*) Allele Arg506Gln and *Prothrombin* (*F2*) Variant G20210A

The role of the Arg506G allele of the *F5* gene (encoding factor V Leiden-F5 Leiden, located on chromosome 1q24.2) and the G20210A allelic variant of the *F2* gene (located on chromosome 11p11.2, encoding prothrombin), in the production of MI, remains controversial. Although the two polymorphisms are associated with an increased risk of venous thrombosis, their role has not been demonstrated in the production of arterial thrombosis. Individuals heterozygous for the *F5* gene Arg506Gln mutation (3–5% of the population) have a seven times higher risk of venous thrombosis, whereas homozygotes have a 100 times higher risk [4].

In the study by Ercan et al. [129], which included 181 patients with angiographically documented CAD and a control group of 107 patients, although the *F5* Arg506G heterozygous mutation and the *F2* G20210A heterozygote mutation were more common in patients with CAD than in the control group, and no statistically significant association was found between their presence and CAD [129]. Most clinical trials have not shown any association between these alleles and the increased risk of CAD/MI, and more studies are needed to reach definitive conclusions [4].

### 6.4. Metabolic Factors: Hyperhomocysteinemia (MTHFR Gene Polymorphism)

The enzyme methylenetetrahydrofolate reductase (MTHFR) encoded by the *MTHFR* gene (located on chromosome 1q36.22) is involved in the remethylation of homocysteine to methionine [16]. The *MTHFR* gene mutations associated with decreased enzymatic activity, will increase plasma homocysteine levels. Because hyperhomocysteinemia is associated with an increased risk of CAD, it has become necessary to study the genes involved in homocysteine metabolism [16,130].

A meta-analysis by Lewis et al. [130], which included 80 studies (26,000 patients and 31,183 controls), showed that there was no solid evidence to support an association of *MTHFR* C677T (rs1801133) polymorphism with CAD in Europe, North America, or Australia. Geographical variability may be due to higher folic acid intake in North America and Europe or poor data communication. Additionally, there is no clear evidence that folic acid administration decreases plasma homocysteine levels, thus having a protective role in the occurrence of cardiovascular disease (CVD) [130]. 

Another meta-analysis by Brattström et al. [131] included 23 studies, and the conclusion was that the *MTHFR* C677T mutation is commonly associated with mild hyperhomocysteinemia and does not increase the risk of CVD [131]. Nedelcu et al. [132] analyzed a group of 61 MI patients under the age of 45, with the results showing a strong association between plasma homocysteine levels and the first MI among young patients, pointing out that plasma homocysteine levels could be a possible risk factor for MI [132].

Klerk et al. [133] analyzed 40 studies (including 11,162 IHD/CAD subjects and 12,758 controls), concluding that *MTHFR* TT polymorphism was associated with an increased risk of IHD in cases associated with folate deficiency. The European population with the *MTHFR* TT genotype had a significantly higher risk of IHD (odds ratio 1.14, 1.01–1.28) compared with North Americans in whom no increase in risk for the same genotype was observed (odds ratio 0.87, 0.73–1.05), the differences being partially explained by the different dietary intake of folates in the two analyzed populations [133].

### 6.5. Genes Associated with Inflammation: IL6 Gene Polymorphism

The *IL6* gene (located on chromosome 7p15.3) encodes interleukin 6 (IL6), a cytokine that regulates the production of C-reactive protein (CRP), an inflammatory marker associated with increased risk of IHD/CAD. The association between *IL6* gene polymorphism and IHD/CAD has been extensively studied. Two common *IL6* gene promoter polymorphisms (-174G>C and -572G>C) were associated in various studies with an increased risk of CAD, in other studies, the results being contradictory [4,80].

In the European HIFMECH study in which patients from two high-risk CAD centers in northern Europe and two low-risk CAD centers in southern Europe were analyzed, the association between *IL6* promoter polymorphisms (-572G>C and -174G>C) with circulating levels of inflammatory markers and the risk of MI. The plasma IL6 and CRP levels were similar in controls in both regions, but were higher in those with CAD. The frequency of the rare -174C allele (-174G>C polymorphism) was higher in the northern European group (0.43 vs. 0.28; *p* < 0.0005), where carriers of the -174C allele had a reduced risk of MI compared with homozygotes -174GG (OR 0.53, 95% CI 0.32, 0.86). This effect was not observed in the southern European population nor in the -572G>C variant (which was not associated with an increase or decrease in the risk of MI). No regional differences of the -572G>C allele frequency were observed. None of the genotypes were associated with a significant effect on plasma IL6 levels, either in patients with CAD or in control groups [134]. 

### 6.6. Other Susceptibility Loci for CAD

GWASs have identified other susceptibility loci for CAD in families with premature CAD, located on chromosomes 1, 2, 3, 14, 16 and X [5,80].

A study that included Icelandic families with peripheral arterial occlusive disease (PAOD) provided evidence of the involvement of the locus on chromosome 1p31 (PAOD locus). The PAOD locus has been associated with stroke and MI caused by atherosclerosis. There was no correlation of the PAOD locus with the occurrence of hyperlipidemia, hypertension, or DM [135].

Linkage analysis in families with premature CAD identified two other chromosomal regions: 2q21.1-q22 and Xq23-q26 [136]. Pajukanta et al. [136] identified the locus 2q21.1-q22 (Coronary Heart Disease, Susceptibility To, 2-CHDS2 locus, OMIM 608316) [16] in a study that included Finnish families, in which the proband showed premature CAD, defined by stenosis with more than 50% of at least two coronary arteries. They later identified the second susceptibility locus for CAD located on the Xq23-q26 chromosome (Coronary heart disease, susceptibility to, 3-CHDS3 locus, OMIM 300464) [16,136]. The angiotensin II receptor 2 (*AGTR2*) gene, located in the Xq32 locus, could play a major role in cardiovascular homeostasis [136]. 

The locus 2q36-q37.3 has been identified by GWAS in families with ACS including MI and unstable angina with onset before the age of 70 years. At this level are located the insulin receptor substrate-1 (*IRS1*) gene, the high-density lipoprotein binding protein (*HDLBP*) gene, and the calpain-10 (*CAPN10*) gene (which determines Non- Insulin Dependent Diabetes Mellitus 1, NIDDM1). Other loci associated with CAD in diabetes mellitus (DM) patients have been located on chromosomes 3q26-q27 and 20q11-q13.82 [5,137]. GWASs in the families of patients with CAD who had coronary artery calcification identified two other loci of interest located on chromosomes 6p21.3 and 10q21.3 [138]. 

Other loci associated with premature CAD were located on chromosomes 3q13, 14q32, and chromosome 16pter-p13 [5]. Zhu et al. [139] showed that the GA/AA polymorphism of the *ALDH2* gene (located on chromosome 12q24) encoding alcohol dehydrogenase 2 (ADH2) is an independent risk factor for MI [139].

## 7. Discussion

A complete understanding of the role of genetic factors in the emergence of CAD is one of the goals of modern medicine. Deciphering the complex pathophysiological mechanisms of CAD, identifying all the genetic factors involved, as well as translating the new information obtained through molecular technology in medical practice, are essential for the development of screening methods based on genetic risk scores. This new approach will facilitate the identification of people at high risk for CAD, the implementation of early prophylactic measures and the establishment of new therapeutic targets. Gene therapy strategies are the next step in the treatment and prevention of the disease.

CAD remains a leading cause of death worldwide, despite improved treatment and prophylactic methods. The most common cause of CAD is coronary atherosclerosis, which can have a monogenic or multifactorial etiology. GWASs and WES discovered over 321 CAD risk loci and many risk genes, most of which are linked to the presence of a disorder of lipid metabolism and hypertension [82]. It is estimated that together, all these genetic factors identified by GWASs explain about 40–60% of CAD heritability, suggesting that there are still unidentified genetic factors [6,85,140]. In addition, possible interactions between different genes (epistasis), mechanisms of epigenetic regulation, as well as interactions between genetic and environmental factors, which cannot be identified by GWASs or WES, should be considered.

### 7.1. Challenges for the Future in the Post-GWAS Era

Identifying all the factors involved is a real challenge for future studies and will certainly contribute to solving this real puzzle represented by the complex genetic architecture of IHD/CAD etiology.

A major challenge in the coming years will be the integration of information from analyses-omics (genomics, epigenomics, transcriptomics, proteomics and metabolomics) and their correlation with phenotypic manifestations (detected by imaging, functional and clinical tests) [10]. Continued progress in this area will depend on the development of new analytical techniques based on large databases. The information obtained will allow a better characterization of specific CAD phenotypes (for each CAD subtype), and the molecular redefinition of the phenotypes will certainly contribute to the development of precision medicine [6,10].

Epidemiological research over the past 50 years has uncovered a multitude of biomarkers often used for CVD risk prediction. However, no conclusions could be drawn to confirm the causal relationship between these biomarkers and CAD, even in the case of strong evidence of their association with the disease.

*Mendelian randomization* (MR) studies may reveal a causal relationship between a biomarker and CAD, providing evidence of the biomarker’s contribution to disease development, specifying whether the observed association is influenced by unrecognized exogenous factors or the disease itself affects the level of the biomarker [10,147,148].

The genetic variant used in this type of study should significantly affect the biomarker investigated, but should not affect other phenotypes that could confound the association between the biomarker and the disease. If this biomarker is a true causal risk factor for CAD, the genotypes of the variant used should be associated with the risk for CAD in the direction predicted by the association of the biomarker with CAD. The analysis of the causal factors of CAD by MR has an extraordinary potential to identify possible therapeutic targets. The opportunities and challenges of MR studies in the case of CAD were discussed, and over time being used several biomarkers involved in lipid metabolism, inflammation, obesity, DM and hypertension [5,10,147,148]. 

The creation of large, accessible international databases and the sharing of information between different study groups will elucidate the contradictory results of some studies that included small groups of patients. Because early-onset CAD appears to be associated with genetic susceptibility more frequently than CAD in elderly patients, future studies should focus on the study of affected young populations. Clarifying ethnic differences in the risk of CAD and the response to different therapies may indicate genetic differences that will allow the development of targeted and personalized drug therapy [4,5].

As the quantity of data provided by different types of studies increases, it will be necessary to improve the methods of statistical and bioinformatics analysis that will help to decipher the complex etiology of IHD/CAD. Candidate gene-based association studies have identified genetic polymorphisms that are significantly associated with a reduced risk of CAD and MI [4,6]. However, the results obtained for many of these potential genetic loci, which would provide protection against CAD, are contradictory. Their number and distribution in the general population are still unknown and their identification will probably be an important objective of future studies [4].

### 7.2. Translating the Results of GWASs into Clinical Practice and the Importance of Polygenic Risk Scores (PRS) for Prevention of CAD

Knowledge of the genetic architecture of CAD has clinical applications such as the identification of new therapeutic targets and the improvement of cardiovascular risk estimation, and in pharmacogenomics [4,10]. GWASs have unequivocally shown that complex common diseases have a polygenic etiology and have allowed researchers to identify genetic variants (polymorphisms) associated with these diseases. These allelic variants can be combined into a polygenic risk score (PRS) that includes some of an individual’s susceptibility to the disease [149].

Improving CVD risk information could be achieved by using polygenic risk scores (PRS) that could establish from birth the existence of a genetic predisposition to CAD [4,10]. Information on genetic factors provides the only tool to guide the prevention of CAD through targeted interventions, before the emergence of traditional cardiovascular risk factors (CRF) or specific clinical manifestations of the disease. Although initially the results were disappointing, later the discovery of numerous polymorphisms associated with CAD, the analysis of large cohorts and the biostatistical processing of the data obtained (as in the case of UK Biobank), allowed the realization of polygenic risk scores (PRS) (at the level of the whole genome) [80]. 

A PRS (polygenic risk score) is calculated as the sum of a several weighted genomic variants to estimate their effect, which was determined by GWAS [80] Many small effect size genetic variants contribute to a person’s susceptibility to CAD. The PRS prediction quantifies the contributing effects in a score and estimates whether the individual tested has a high and medium risk of CAD [149]. PRS is the aggregate contribution of many common genetic variants (minor alleles with a frequency > 0.01) that have small to moderate individual effects. The PRS are singular, quantitative values for genetic susceptibility to polygenic diseases such as CAD. GWAS demonstrates that several common genetic variants make individuals susceptible to CAD. GWASs allow the systematic and individual comparison of the prevalence of SNPs in individuals with CAD and those without CAD, to generate SNP-level association statistics, these statistics being the central element of PRS [150]. Initially, most PRS for CAD were made for the European population and could not be used for people of other ethnicities. Identifying these ethnic genetic differences was a challenge, along with identifying new genetic risk factors for CAD, in the era of genomic medicine [80].

Subsequently, the predictive power of PRS for CAD has been improved by including evidence of association, linkage disequilibrium, anticipated functional impact, pleiotropy, and cross-ancestral data that allow the use of PRS in populations of different ethnicities (diverse ancestry). In the case of CAD, PRS could be used to identify high-risk individuals who would benefit from early prophylactic measures through intensive lifestyle modification, imaging surveillance and early lipid-lowering therapy (statins). Completed clinical trials have shown that individuals with a high PRS for CAD obtain the maximum benefits from early LDL-C lowering therapy [150].

### 7.3. Prophylactic Measures in Families at High Risk for CAD

Knowing the genetic risk factors of CAD in certain groups of patients requires customizing prevention and treatment strategies. In their case, adopting a healthy lifestyle with a balanced diet and regular exercise and avoiding traditional CAD risk factors such as excessive lipid consumption and smoking, could reduce the severity of the disease and the onset of acute complications such as MI. The use of risk assessment guidelines as well as the approach of personalized prevention strategies based on family risk is a common practice in the management of patients/families at high risk of CAD [5,14,80]. 

### 7.4. Genetic Counseling in Families at High Risk for CAD

Because the disease can have a monogenic etiology (especially in atherosclerotic CAD) or a complex, multifactorial, or polygenic inheritance, the calculation of disease risk and genetic counseling in high-risk CAD families is based on the genetic risk factors present in that family.

Genetic counseling for patients at high risk for CAD should include, in addition to detailed physical examination, family history and pedigree analysis (which may provide important information on family aggregation or for a monogenic inheritance), personal medical history, habits, and medicines used [5,14].

Disease risk assessment and genetic counseling can be difficult because in most cases CAD has a multifactorial etiology and a large number of loci and genes are involved. Moreover, to this is added the variable interaction between different genes (epistasis) as well as the interaction between genetic and environmental factors.

Although we focused on an in-depth analysis of data from the literature on the role of CAD genetic factors, as well as methods of their analysis, we still consider that our study was limited by incomplete data from the literature on CAD etiology (the “missing heritability” of CAD). Deciphering the etiology of CAD/CHD is a fascinating topic, which remains relevant due to the complexity of the possible factors involved and the interaction between them. In addition, future research will most likely identify new CAD candidate biomarkers, which, together with the use of PRS, will improve CAD risk prediction.

## 8. Conclusions

In the last decade, remarkable progress has been made in elucidating the complex etiology of CAD with the help of increasingly advanced molecular technologies and the processing of data obtained by efficient statistical and bioinformatics methods. Although overall CAD mortality has remained high, the main benefit has been the identification of new therapeutic targets. 

In addition, GWASs provided information that offered a new perspective on the complex pathophysiology of CAD, and the newly identified genetic factors, along with those already known, currently represent about 40–50% of CAD heritability.

Future identification of new genetic factors could explain the “missing heritability” of CAD, without ignoring the fact that the interaction between different genes or between genetic and environmental factors could determine the phenotypic variability of CAD. Additionally, the identification of genetic factors that would have a protective role against CAD may be an important objective of future research.

The current use of PRS could improve CAD risk prediction, allowing the identification of people at higher risk for CAD, who could benefit from personalized prevention and treatment.

It is expected that following the sustained efforts of the large international consortiums, new genetic risk factors for CAD will be identified, with translation into clinical practice related to the development of new therapeutic molecules that act on specific targets.

## Figures and Tables

**Table 1:** Genetic etiology of coronary artery disease.

Genetic Transmission Monogenic/Polygenic	Gene(s)	Location/ Chromosome	Disease/CAD-S	Biochemical Changes	Method CGS/GWAS/LA/WES	References
**Monogenic lipid CAD**						
	*LDLR*	19p13.2	FH	↑ LDL-C	CGS/GWAS	[4,6,16,17,18]
	*APOB*	2p24.1	FHCL2/FDB	↑ LDL-C	CGS/GWAS	[4,16,19,20,21,22]
	*PCSK9*	1p34.1-p32	HCHOLA3	↑ LDL-C	CGS/GWAS	[16,23,24,25,26,27]
	*LDLRAP1*	1p34-p35	ARH	↑ LDL-C	CGS	[16,28,29]
	*APOAI*	11q23.3	Apo AI deficiency, Apo-A1 and apo C-III combined deficiency	↓ HDL-C	CGS	[4,16,30,31,32,33,34]
	*ABCA1*	9q31.1	TGD	↓ HDL-C	CGS/GWAS	[16,35,36,37]
	*LCAT*	16q22.1	LCAT deficiency	↓ HDL-C	CGS/GWAS	[10,16,38,39]
	*LPL*	8p21.3	LPL deficiency/CHLF	↑ TG, ↓ LDL-C, ↓ HDL-C	CGS	[16,40,41]
	*APOC2*	19q13.2	HL Ib	↑ TG	CGS	[16,42]
	*ABCG5*, *ABCG8*	2p21	STSL	↑ plant sterols	CGS	[6,43,44]
	*APOA1/C3/A4/A5*	11p14.1-q12.1, 1q21-23, 16q22-24.1	FCHL	↑ VLDL, ↑ LDL-C, ↑ ApoB, ↑ TG	LA/GWAS	[6,45,46,47,48,49,50]
	*APOA5*	11p14.1-q12.1, 15q11.2-q13.1, 8q11-q13	FHTG	↑ TG	LA/GWAS	[4,16,50,51,52,53]
	*ATHS*	19p13.3-p13.2	ATHS/ALP	↑ LDL-C, ↑ TG, ↓ HDL-C	LA	[16,54,55,56,57,58]
**Other Monogenic CAD**						
	*MEF2A*	15q26	ADCAD1		CGS	[6,59,60,61,62]
	*ST6GALNAC5*	1p31.1			GWAS/WES	[16,63,64]
	*CYP27A1*	2p35		↓ LDL, ↓ VLDL, ↑ HDL-C		
		
			CTX		WES	[16,65,66,67,68]
	*LRP6*	12p13.2		↑ LDL-C, ↑ TG	CGS/GWAS	[16,69,70]
**Polygenic CAD**						
	*CDKN2A*, *CDKN2B*	9p21	CAD-S		GWAS	[8,10,71,72,73,74]
	*C6orf105 gene*	6p24.1	CAD-S		GWAS	[6,75,76]
	*COL4A1/COL4A2*, *ZC3HC1*, *CYP17A1*	13q34, 7q32.2, 10q24.32	CAD-S		GWAS	[77,78]
	*CTSS*	1q21	CAD-S		GWAS	[79]
	*WDR11-FGFR2*	10q26	CAD-S		GWAS	[79]
	*RDX-FDX1*	11q22	CAD-S		GWAS	[79]
	*PSRC1*	1p13.3	CAD-S		GWAS	[73,80]
	*MIA3*	1q41	CAD-S		GWAS	[73,80]
	*SMAD3*	15q22.3	CAD-S		GWAS	[73,81,82,83]
Polygenic lipid CAD	*APOE*,	19q13.32,	CAD-S		GWAS	[84,85,86,87,88,89,90,91]
	*APOB*,	2p24.1,				[6,21,92,93,94]
	*LPL*,	8p21.3,				[80,95,96,97,98]
	*OLR1 (LOX1)*,	12p13.2,				[99,100]
	*SORT1*,	1p13.3,				[101]
	*TRIB1*,	8q24.13				[80,102,103]
Genes associated with vascular homeostasis	*NOS3*,	7q36.1,	CAD-S		GWAS	[4,104,105,106]
	*TCF21*,	6q23.2,				[80,107,108]
	*ADAMTS7*,	15q25.1,				[80,109]
	*HHIPL1*,	14q32,				[110]
	*ACE*,	17q23.3,				[80,111,112,113,114]
	*AGT*,	1q42.2,				[114]
	*AGT1R*,	3q23,				[115,116]
	*CYP11B2*	8q24.3				[117,118,119]
Genes associated with vascular hemostasis	*ITGA2*,	5q11.2,	CAD-S		GWAS	[4,120,121]
	*ITGB2*,	21q22.3,				[122,123]
	*ITGB3*,	17q21.32,				[124,125]
	*PAI-1*,	7q22.1,				[126,127]
	*THBS (1*, *2*, *4)*,	15q14, 6q27, 5q14.1				[128]
	*F5 Leiden (Arg506Gln)*,	1q24.2,				[4,129]
	*F2 gene (Arg 506Gln)*	11p11.2				[4,129]
HHcy	*MTHFR*	1p36.22	CAD-S		GWAS	[130,131,132,133]
Inflammation	*IL-6*	7p15.3	CAD-S		GWAS	[4,80,134]
Other genes	*POAD*	1p31	CAD-S		GWAS	[135]
	*CHDS2*,	2q21.2-q22,				[136]
	*CHDS3*,	Xq23-q26,				[16,136]
	*AGTR2*,	Xq23,				[136]
	*IRS1*, *CAPN10*,	2q36-q37.3				[5,137,138]
	*HDLBPALDH2*	12q24				[139]

CAD: Coronary artery disease; MI: Acute myocardial infarction; HDL-C: High-density lipoprotein cholesterol; LDL-C: Low-density lipoprotein cholesterol; TG: Triglyceride; CGS: Candidate gene-based association studies; GWAS: Genome-wide association studies; LA: Genetic Linkage analysis; WES: Whole exome sequencing; FH: Familial hypercholesterolemia; FDB: Familial defective apolipoprotein B-100; FHCL2: Hypercholesterolemia, familial, 2; HCHOLA3: Hypercholesterolemia, autosomal dominant, 3; ARH: Autosomal recessive hypercholesterolemia; TGD: Tangier disease; HLIb: Hyperlipoproteinemia type Ib; LPL deficiency: Lipoprotein lipase deficiency; CHLF: Combined hyperlipidemia, familial; STSL: Sitosterolemia; FCHL: Familial combined hyperlipidemia; FHTG: Familial hypertriglyceridemia; CTX: Cerebrotendinous Xanthomatosis; VLDL: Very low-density lipoprotein; APOB: Apolipoprotein B; Atherosclerosis Susceptibility (ATHS)/Atherogenic Lipoprotein Phenotype (ALP); ADCAD1: Coronary artery disease, autosomal dominant, 1; F5 Leiden: Factor V Leiden; F2 gene: prothrombin; CAD-S: CAD Susceptibility; HHcy: Hyperhomocysteinemia.

**Table 2:** Candidate genes and CAD-associated genetic polymorphisms identified by GWASs [10,71,75,78,81,83].

Location/Chromosome	Gene (s)	SNPs	Risk Allele	Risk Allele Frequency
1p32.3	*PCSK9*	rs112065101	T/C	0.848
1p32.3	*PPAP2B*	rs9970807	C/T	0.915
1p13.3	*SORT1*	rs7528419	A/G	0.786
1q21	*CTSS*	rs6587520	T/C	0.480
1q21.3	*IL6R*	rs6689306	A/G	0.448
1q41	*MIA3*	rs67180937	G/T	0.663
2p24.1	*AK097927*	rs16986953	A/G	0.105
2p24.1	*APOB*	chr2:21378433:D	D/I	0.746
2p21	*ABCG5, ABCG8*	chr2:44074126:D	I/D	0.745
2p11.2	*VAMP5-VAMP8-GCX*	rs7568458	A/T	0.449
2q22.3	*ZEB2-ACO74093.1*	rs17678683	G/T	0.088
2q33.2	*WDR12*	chr2:203828796:I	I/D	0.108
3q22.3	*MRAS*	chr3:138099161:I	I/D	0.163
4q31.22-q31.23	*EDNRA*	rs4593108	C/G	0.795
4q32.1	*GUCY1A3*	rs72689147	G/T	0.817
4q12	*REST-NOA1*	rs17087335	T/G	0.210
5q31.1	*SLC22A4-SLC22A5*	rs273909	G/A	0.117
6p24.1	*ADTRP-C6orf105*	rs6903956	A/G	0.354
6p24.1	*PHACTR1*	rs9349379	G/A	0.432
6p21.31	*ANKS1A*	rs17609940	G/C	0.824
6p21.2	*KCNK5*	rs56336142	T/C	0.807
6q23.2	*TCF21*	rs12202017	A/G	0.700
6q25.3	*SLC22A3-LPAL2-LPA*	rs55730499	T/C	0.056
6q26	*PLG*	rs4252185	C/T	0.060
7p21.1	*HDAC9*	rs2107595	A/G	0.200
7q22.3	*BCAP29*	rs10953541	C/T	0.783
7q34	*ZC3HC1 (PARP12)*	rs11556924	C/T	0.687
7q36.1	*NOS3*	rs17087335	T/C	0.060
8p21.3	*LPL*	rs264	G/A	0.853
8q24.13	*TRIB1*	rs2954029	A/T	0.551
9p21.3	*CDKN2BAS*	rs2891168	G/A	0.489
9q34.2	*ABO*	rs2519093	T/C	0.191
10p11.23	*KIAA1462*	rs2487928	A/G	0.418
10q11.21	*CXCL12*	rs1870634	G/T	0.637
10q23.31	*LIPA*	rs1412444	T/C	0.369
10q24.32	*CYP17A1-CNNM2-NT5C2*	rs11191416	T/G	0.873
10q26	*WDR11-FGFR2*	rs2257129	C/T	0.900
11q22.3	*PDGFD*	rs2128739	A/C	0.324
11q22	*RDX-FDX1*	rs10488763	T/A	0.180
11q23.3	*ZNF259-APOA5-APOA1*	rs964184	G/C	0.185
11p15.4	*SWAP70*	rs10840293	A/G	0.550
12q21.33	*ATP2B1*	rs2681472	G/A	0.201
12q24.12	*SH2B3*	rs3184504	T/C	0.422
12q24.22-q24.23	*KSR2*	rs1180803	G/T	0.360
13q12.3	*FLT1*	rs9319428	A/G	0.314
13q34	*COL4A1-COL4A2*	rs11838776	A/G	0.263
14q32	*HHIPL1*	rs10139550	G/C	0.423
15q25.1	*ADAMTS7*	rs4468572	C/T	0.586
15q26.1	*FURIN-FES*	rs17514846	A/C	0.440
15q22.33	*SMAD3*	rs56062135	C/T	0.790
15q26.1	*MFGE8-ABHD2*	rs8042271	G/A	0.900
17p13.3	*SMG6*	rs216172	C/G	0.350
17p11.2	*RAI1-PEMT-RASD1*	rs12936587	G/A	0.611
17q21.32	*UBE2Z*	rs46522	T/C	0.513
17q23.2	*BCAS3*	rs7212798	C/T	0.150
18q21.32	*PMAIP1-MC4R*	rs663129	A/G	0.260
19p13.2	*LDLR*	rs56289821	G/A	0.900
19q13.32	*APOE-APOC1*	rs4420638	G/A	0.166
19q13.11	*ZNF507-LOC400684*	rs12976411	T/A	0.090
21q22.11	*KCNE2*	rs28451064	A/G	0.121
22q11.23	*POM121L9P-ADORA2A*	rs180803	G/T	0.970

CAD: Coronary artery disease; A-adenine; C-cytosine; G-guanine; T-thymine; D-deletion; I-insertion; SNP-Single nucleotide polymorphism.

## Data Availability

Not applicable.

## Abbreviations

CAD: Coronary artery disease; IHD: Ischemic heart disease; MI: Acute myocardial infarction; ACS: Acute coronary syndrome; SCD: Sudden cardiac death; PA: Angina pectoris (myocardial ischaemia); CVD: Cardiovascular disease; CRF: Cardiovascular risk factors; DM: Diabetes mellitus; T2DM: Type 2 diabetes mellitus; PRS: Polygenic risk scores; TC: Total cholesterol; VSMc: Vascular smooth muscle cells; MAF: Minor allele frequency; HDL-C: High-density lipoprotein cholesterol; LDL-C: Low-density lipoprotein cholesterol; TG: Triglyceride; CGS: Candidate gene-based association studies; GWAS: Genome-wide association studies; LA: Genetic Linkage analysis; WES: Whole exome sequencing.

## References

[1] European Heart References Network (EHN) (2017). European Cardiovascular Disease Statistics. https://ehnheart.org/cvd-statistics.html/.

[2] Heart Disease Statistics 2022 Gerardo Sison Editor. https://www.singlecare.com/blog/news/heart-disease-statistics/.

[3] Muse E.D., Chen S.F., Torkamani A. (2021). Monogenic and Polygenic Models of Coronary Artery Disease. Curr. Cardiol. Rep..

[4] Nordlie M.A., Wold L.E., Kloner R.A. (2005). Genetic contributors toward increased risk for ischemic heart disease. J. Mol. Cell. Cardiol..

[5] Scheuner M.T. (2003). Genetic evaluation for coronary artery disease. Genet. Med..

[6] Dai X., Wiernek S., Evans J.P., Runge M.S. (2016). Genetics of coronary artery disease and myocardial infarction. World J. Cardiol..

[7] Mach F., Baigent C., Catapano A.L., Koskinas K.C., Casula M., Badimon L., Chapman M.J., De Backer G.G., Delgado V., Ference B.A. (2020). ESC Scientific Document Group. 2019 ESC/EAS Guidelines for the Management of Dyslipidaemias: Lipid Modification to Reduce Cardiovascular Risk. Eur. Heart J..

[8] Visseren F.L.J., Mach F., Smulders Y.M., Carballo D., Koskinas K.C., Bäck M., Benetos A., Biffi A., Boavida J.M., Capodanno D. (2022). ESC Scientific Document Group. 2021 ESC Guidelines on cardiovascular disease prevention in clinical practice: Developed by the Task Force for cardiovascular disease prevention in clinical practice with representatives of the European Society of Cardiology and 12 medical societies with the special contribution of the European Association of Preventive Cardiology (EAPC). Rev. Esp. Cardiol..

[9] Fedele F., Pucci M., Severino P., Parine N.R. (2017). Genetic Polymorphisms and Ischemic Heart Disease. Genetic Polymorphisms.

[10] Elosua R., Sayols-Baixeras S. (2017). The Genetics of Ischemic Heart Disease: From Current Knowledge to Clinical Implications. Rev. Esp. Cardiol..

[11] Whelton P.K., Carey R.M., Aronow W.S., Casey D.E., Collins K.J., Dennison Himmelfarb C., DePalma S.M., Gidding S., Jamerson K.A., Jones D.W. (2018). 2017 ACC/AHA/AAPA/ABC/ACPM/AGS/APhA/ASH/ASPC/NMA/PCNA Guideline for the Prevention, Detection, Evaluation, and Management of High Blood Pressure in Adults: Executive Summary: A Report of the American College of Cardiology/American Heart Association Task Force on Clinical Practice Guidelines. Hypertension.

[12] Yusuf S., Hawken S., Ounpuu S., Dans T., Avezum A., Lanas F., McQueen M., Budaj A., Pais P., Varigos J. (2004). Effect of potentially modifiable risk factors associated with myocardial infarction in 52 countries (the INTERHEART study): Case-control study. Lancet.

[13] Pechlivanis S., Lehmann N., Hoffmann P., Nöthen M.M., Jöckel K.H., Erbel R., Moebus S. (2020). Risk prediction for coronary heart disease by a genetic risk score—Results from the Heinz Nixdorf Recall study. BMC Med. Genet..

[14] Knuuti J., Wijns W., Saraste A., Capodanno D., Barbato E., Funck-Brentano C., Prescott E., Storey R.F., Deaton C., Cuisset T. (2020). ESC Scientific Document Group. 2019 ESC Guidelines for the diagnosis and management of chronic coronary syndromes. Eur. Heart J..

[15] Brown J.C., Gerhardt T.E., Kwon E. (2021). Risk Factors for Coronary Artery Disease. StatPearls.

[16] OMIM—Online Mendelian Inheritance in Man. https://www.omim.org.

[17] Gabcova-Balaziova D., Stanikova D., Vohnout B., Huckova M., Stanik J., Klimes I., Raslova K., Gasperikova D. (2015). Molecular-genetic aspects of familial hypercholesterolemia. Endocr. Regul..

[18] Abifadel M.S., Rabès J.H., Boileau C.R. (2021). Genetic Testing in Familial Hypercholesterolemia: Strengthening the Tools, Reinforcing Efforts, and Diagnosis. JACC Basic Transl. Sci..

[19] Soria L.F., Ludwig E.H., Clarke H.R., Vega G.L., Grundy S.M., McCarthy B.J. (1989). Association between a specific apolipoprotein B mutation and familial defective apolipoprotein B-100. Proc. Natl. Acad. Sci. USA.

[20] Andersen L.H., Miserez A.R., Ahmad Z., Andersen R.L. (2016). Familial defective apolipoprotein B-100: A review. J. Clin. Lipidol..

[21] Pullinger C.R., Hennessy L.K., Chatterton J.E., Liu W., Love J.A., Mendel C.M., Frost P.H., Malloy M.J., Schumaker V.N., Kane J.P. (1995). Familial ligand-defective apolipoprotein B. Identification of a new mutation that decreases LDL receptor binding affinity. J. Clin. Investig..

[22] Thomas E.R., Atanur S.S., Norsworthy P.J., Encheva V., Snijders A.P., Game L., Vandrovcova J., Siddiq A., Seed M., Soutar A.K. (2013). Identification and biochemical analysis of a novel APOB mutation that causes autosomal dominant hypercholesterolemia. Mol. Genet. Genom. Med..

[23] Schmidt R.J., Beyer T.P., Bensch W.R., Qian Y.-W., Lin A., Kowala M., Alborn W.E., Konrad R.J., Cao G. (2008). Secreted proprotein convertase subtilisin/kexin type 9 reduces both hepatic and extrahepatic low-density lipoprotein receptors in vivo. Biochem. Biophys. Res. Commun..

[24] Abifadel M., Rabès J.P., Devillers M., Munnich A., Erlich D., Junien C., Varret M., Boileau C. (2009). Mutations and polymorphisms in the proprotein convertase subtilisin kexin 9 (PCSK9) gene in cholesterol metabolism and disease. Hum. Mutat..

[25] Kathiresan S., Voight B.F., Purcell S., Musunuru K., Ardissino D., Mannucci P.M., Anand S., Engert J.C., Samani N.J., Schunkert H. (2009). Genome-wide association of early-onset myocardial infarction with single nucleotide polymorphisms and copy number variants. Nat. Genet..

[26] Cohen J.C., Boerwinkle E., Mosley T.H., Hobbs H.H. (2006). Sequence variations in PCSK9, low LDL, and protection against coronary heart disease. N. Engl. J. Med..

[27] Chuan J., Qian Z., Zhang Y., Tong R., Peng M. (2019). The association of the PCSK9 rs562556 polymorphism with serum lipids level: A meta-analysis. Lipids Health Dis..

[28] D’Erasmo L., Di Costanzo A., Arca M. (2020). Autosomal recessive hypercholesterolemia: Update for 2020. Curr. Opin. Lipidol..

[29] Arca M., Zuliani G., Wilund K., Campagna F., Fellin R., Bertolini S., Calandra S., Ricci G., Glorioso N., Maioli M. (2002). Autosomal recessive hypercholesterolaemia in Sardinia, Italy, and mutations in ARH: A clinical and molecular genetic analysis. Lancet.

[30] Novo S. (2009). Low HDL-cholesterol concentrations cause atherosclerotic disease to develop. ESC European Society of Cardiology. E-J. ESC Counc. Cardiol. Pract..

[31] Rader D.J., deGoma E.M. (2012). Approach to the patient with extremely low HDL-cholesterol. J. Clin. Endocrinol. Metab..

[32] Yamakawa-Kobayashi K., Yanagi H., Fukayama H., Hirano C., Shimakura Y., Yamamoto N., Arinami T., Tsuchiya S., Hamaguchi H. (1999). Frequent occurrence of hypoalphalipoproteinemia due to mutant apolipoprotein A-I gene in the population: A population-based survey. Hum. Molec. Genet..

[33] Haase C.L., Frikke-Schmidt R., Nordestgaard B.G., Tybjærg-Hansen A. (2012). Population-based resequencing of APOA1 in 10,330 individuals: Spectrum of genetic variation, phenotype, and comparison with extreme phenotype approach. PLoS Genet..

[34] Bielicki J.K., Oda M.N. (2002). Apolipoprotein A-I(Milano) and apolipoprotein A-I(Paris) exhibit an antioxidant activity distinct from that of wild-type apolipoprotein A-I. Biochemistry.

[35] Koseki M., Yamashita S., Ogura M., Ishigaki Y., Ono K., Tsukamoto K., Hori M., Matsuki K., Yokoyama S., Harada-Shiba M. (2021). Current Diagnosis and Management of Tangier Disease. J. Atheroscler. Thromb..

[36] The Human Gene Mutation Database at the Institute of Medical Genetics in Cardiff. http://www.hgmd.cf.ac.uk/ac/gene.php?gene=ABCA1/.

[37] Mokuno J., Hishida A., Morita E., Sasakabe T., Hattori Y., Suma S., Okada R., Kawai S., Naito M., Wakai K. (2015). ATP-binding cassette transporter A1 (ABCA1) R219K (G1051A, rs2230806) polymorphism and serum high-density lipoprotein cholesterol levels in a large Japanese population: Cross-sectional data from the Daiko Study. Endocr. J..

[38] Mehta R., Elías-López D., Martagón A.J., Pérez-Méndez O.A., Sánchez M.L.O., Segura Y., Tusié M., Aguilar-Salinas C.A. (2021). LCAT deficiency: A systematic review with the clinical and genetic description of Mexican kindred. Lipids Health Dis..

[39] McIntyre N. (1988). Familial LCAT deficiency and fish-eye disease. J. Inherit. Metab. Dis..

[40] Reiner Ž. (2017). Hypertriglyceridaemia and risk of coronary artery disease. Nat. Rev. Cardiol..

[41] Gehrisch S. (1999). Common mutations of the lipoprotein lipase gene and their clinical significance. Curr. Atheroscler. Rep..

[42] Cox D.W., Breckenridge W.C., Little J.A. (1978). Inheritance of apolipoprotein C-II deficiency with hypertriglyceridemia and pancreatitis. N. Engl. J. Med..

[43] Yoo E.G. (2016). Sitosterolemia: A review and update of pathophysiology, clinical spectrum, diagnosis, and management. Ann. Pediatr. Endocrinol. Metab..

[44] Wang Z., Caom L., Sum Y., Wang G., Wang R., Yu Z., Bai X., Ruan C. (2014). Specific macrothrombocytopenia/hemolytic anemia associated with sitosterolemia. Am. J. Hematol..

[45] Liu Y., Ordovas J.M., Gao G., Province M., Straka R.J., Tsai M.Y., Lai C.Q., Zhang K., Borecki I., Hixson J.E. (2009). Pharmacogenetic association of the APOA1/C3/A4/A5 gene cluster and lipid responses to fenofibrate: The genetics of lipid-lowering drugs and diet network study. Pharm. Genom..

[46] Eichenbaum-Voline S., Olivier M., Jones E.L., Naoumova R.P., Jones B., Gau B., Patel H.N., Seed M., Betteridge D.J., Galton D.J. (2004). Linkage and association between distinct variants of the APOA1/C3/A4/A5 gene cluster and familial combined hyperlipidemia. Arter. Thromb. Vasc. Biol..

[47] Timpson N.J., Walter K., Min J.L., Tachmazidou I., Malerba G., Shin S.Y., Chen L., Futema M., Southam L., Iotchkova V. (2014). UK1OK Consortium Members. A rare variant in APOC3 is associated with plasma triglyceride and VLDL levels in Europeans. Nat. Commun..

[48] Taghizadeh E., Esfehani R.J., Sahebkar A., Parizadeh S.M., Rostami D., Mirinezhad M., Poursheikhani A., Mobarhan M.G., Pasdar A. (2019). Familial combined hyperlipidemia: An overview of the underlying molecular mechanisms and therapeutic strategies. IUBMB Life.

[49] Hopkins P.N., Heiss G., Ellison R.C., Province M.A., Pankow J.S., Eckfeldt J.H., Hunt S.C. (2003). Coronary artery disease risk in familial combined hyperlipidemia and familial hypertriglyceridemia: A case-control comparison from the National Heart, Lung, and Blood Institute Family Heart Study. Circulation.

[50] Bogari N.M., Aljohani A., Amin A.A., Al-Allaf F.A., Dannoun A., Taher M.M., Elsayed A., Rednah D.I., Elkhatee O., Porqueddu M. (2019). A genetic variant c.553G>T (rs2075291) in the apolipoprotein A5 gene is associated with altered triglycerides levels in coronary artery disease (CAD) patients with lipid lowering drug. BMC Cardiovasc. Disord..

[51] Kao J.T., Wen H.C., Chien K.L., Hsu H.C., Lin S.W. (2003). A novel genetic variant in the apolipoprotein A5 gene is associated with hypertriglyceridemia. Hum. Mol. Genet..

[52] Do R., Project N.E.S., Stitziel N., Won H.-H., Jørgensen A.B., Duga S., Merlini P.A., Kiezun A., Farrall M., NHLBI Exome Sequencing Project (2015). Exome sequencing identifies rare LDLR and APOA5 alleles conferring risk for myocardial infarction. Nature.

[53] Genest J.J., Martin-Munley S.S., McNamara J.R., Ordovas J.M., Jenner J., Myers R.H., Silberman S.R., Wilson P.W., Salem D.N., Schaefer E.J. (1992). Familial lipoprotein disorders in patients with premature coronary artery disease. Circulation.

[54] Austin M.A., Breslow J.L., Hennekens C.H., Buring J.E., Willett W.C., Krauss R.M. (1988). Low-Density Lipoprotein Subclass Patterns and Risk of Myocardial Infarction. JAMA.

[55] Nishina P.M., Johnson J.P., Naggert J.K., Krauss R.M. (1992). Linkage of atherogenic lipoprotein phenotype to the low density lipoprotein receptor locus on the short arm of chromosome 19. Proc. Natl. Acad. Sci. USA.

[56] Rotter J.I., Bu X., Cantor R.M., Warden C.H., Brown J., Gray R.J., Blanche P.J., Krauss R.M., Lusis A.J. (1996). Multilocus genetic determinants of LDL particle size in coronary artery disease families. Am. J. Hum. Genet..

[57] Srisawasdi P., Rodcharoen P., Vanavanan S., Chittamma A., Sukasem C., Na Nakorn C., Dejthevaporn C., Kroll M.H. (2021). Association of CETP Gene Variants with Atherogenic Dyslipidemia Among Thai Patients Treated with Statin. Pharmgenom. Pers. Med..

[58] Allayee H., Aouizerat B.E., Cantor R.M., Dallinga-Thie G.M., Krauss R.M., Lanning C.D., Rotter J.I., Lusis A.J., de Bruin T.W. (1998). Families with familial combined hyperlipidemia and families enriched for coronary artery disease share genetic determinants for the atherogenic lipoprotein phenotype. Am. J. Hum. Genet..

[59] Omidi S., Ghasemi S., Kalayinia S. (2020). Is There Any Association Between the MEF2A Gene Changes and Coronary Artery Disease?. Acta Med. Iran..

[60] Wang L., Fan C., Topol S.E., Topol E.J., Wang Q. (2003). Mutation of MEF2A in an inherited disorder with features of coronary artery disease. Science.

[61] Huang X.C., Wang W. (2015). Association of MEF2A gene 3’UTR mutations with coronary artery disease. Genet. Mol. Res..

[62] Liu B., Pang L., Ji Y., Fang L., Tian C.W., Chen J., Chen C., Zhong Y., Ou W.C., Xiong Y. (2022). MEF2A Is the Trigger of Resveratrol Exerting Protection on Vascular Endothelial Cell. Front. Cardiovasc. Med..

[63] InanlooRahatloo K., Parsa A.F., Huse K., Rasooli P., Davaran S., Platzer M., Kramer M., Fan J.B., Turk C., Amini S. (2014). Mutation in ST6GALNAC5 identified in family with coronary artery disease. Sci. Rep..

[64] Cheeseman J., Kuhnle G., Stafford G., Gardner R.A., Spencer D.I., Osborn H.M. (2021). Sialic acid as a potential biomarker for cardiovascular disease, diabetes and cancer. Biomark. Med..

[65] Inanloorahatloo K., Zand Parsa A.F., Huse K., Rasooli P., Davaran S., Platzer M., Fan J.B., Amini S., Steemers F., Elahi E. (2013). Mutation in CYP27A1 identified in family with coronary artery disease. Eur. J. Med. Genet..

[66] Chen C., Zhang Y., Wu H., Sun Y.M., Cai Y.H., Wu J.J., Wang J., Gong L.Y., Ding Z.T. (2017). Clinical and molecular genetic features of cerebrotendinous xanthomatosis patients in Chinese families. Metab. Brain Dis..

[67] Lee C.W., Lee J.J., Lee Y.F., Wang P.W., Pan T.L., Chang W.N., Tsai M.H. (2019). Clinical and molecular genetic features of cerebrotendinous xanthomatosis in Taiwan: Report of a novel CYP27A1 mutation and literature review. J. Clin. Lipidol..

[68] Rashvand Z., Kahrizi K., Najmabadi H., Najafipour R., Omrani M.D. (2021). Clinical and Genetic Characteristics of Splicing Variant in CYP27A1 in an Iranian Family with Cerebrotendinous xanthomatosis. Iran. Biomed. J..

[69] Mani A., Radhakrishnan J., Wang H., Mani A., Mani M.A., Nelson-Williams C., Carew K.S., Mane S., Najmabadi H., Wu D. (2007). LRP6 mutation in a family with early coronary disease and metabolic risk factors. Science.

[70] Wang H., Liu Q.J., Chen M.Z., Li L., Zhang K., Cheng G.H., Ma L., Gong Y.Q. (2012). Association of common polymorphisms in the LRP6 gene with sporadic coronary artery disease in a Chinese population. Chin. Med. J..

[71] McPherson R., Pertsemlidis A., Kavaslar N., Stewart A., Roberts R., Cox D.R., Hinds D.A., Pennacchio L.A., Tybjaerg-Hansen A., Folsom A.R. (2007). A common allele on chromosome 9 associated with coronary heart disease. Science.

[72] Helgadottir A., Thorleifsson G., Manolescu A., Gretarsdottir S., Blondal T., Jonasdottir A., Jonasdottir A., Sigurdsson A., Baker A., Palsson A. (2007). A common variant on chromosome 9p21 affects the risk of myocardial infarction. Science.

[73] Samani N.J., Erdmann J., Hall A.S., Hengstenberg C., Mangino M., Mayer B., Dixon R.J., Meitinger T., Braund P., Wichmann H.E. (2007). WTCCC and the Cardiogenics Consortium Genomewide association analysis of coronary artery disease. N. Engl. J. Med..

[74] Nikpay M., Goel A., Won H.H., Hall L.M., Willenborg C., Kanoni S., Saleheen D., Kyriakou T., Nelson C.P., Hopewell J.C. (2015). A comprehensive 1,000 Genomes-based genome-wide association meta-analysis of coronary artery disease. Nat. Genet..

[75] Deloukas P., Kanoni S., Willenborg C., Farrall M., Assimes T.L., Thompson J.R., Ingelsson E., Saleheen D., Erdmann J., Goldstein B.A. (2013). Large-scale association analysis identifies new risk loci for coronary artery disease. Nat. Genet..

[76] Wang F., Xu C.Q., He Q., Cai J.P., Li X.C., Wang D., Xiong X., Liao Y.H., Zeng Q.T., Yang Y.Z. (2011). Genome-wide association identifies a susceptibility locus for coronary artery disease in the Chinese Han population. Nat. Genet..

[77] IBC 50K CAD Consortium (2011). Large-scale gene-centric analysis identifies novel variants for coronary artery disease. PLoS Genet..

[78] Koyama S., Ito K., Terao C., Akiyama M., Horikoshi M., Momozawa Y., Matsunaga H., Ieki H., Ozaki K., Onouchi Y. (2020). Population-specific and trans-ancestry genome-wide analyses identify distinct and shared genetic risk loci for coronary artery disease. Population-specific and trans-ancestry genome-wide analyses identify distinct and shared genetic risk loci for coronary artery disease. Nat. Genet..

[79] Matsunaga H., Ito K., Akiyama M., Takahashi A., Koyama S., Nomura S., Ieki H., Ozaki K., Onouchi Y., Sakaue S. (2020). Transethnic Meta-Analysis of Genome-Wide Association Studies Identifies Three New Loci and Characterizes Population-Specific Differences for Coronary Artery Disease. Circ. Genom. Precis. Med..

[80] Kessler T., Schunkert H. (2021). Coronary Artery Disease Genetics Enlightened by Genome-Wide Association Studies. JACC Basic Transl. Sci..

[81] Shadrina A.S., Shashkova T.I., Torgasheva A.A., Sharapov S.Z., Klarić L., Pakhomov E.D., Alexeev D.G., Wilson J.F., Tsepilov Y.A., Joshi P.K. (2020). Prioritization of causal genes for coronary artery disease based on cumulative evidence from experimental and in silico studies. Sci. Rep..

[82] Chen Z., Schunkert H. (2021). Genetics of coronary artery disease in the post-GWAS era. J. Intern. Med..

[83] LeBlanc M., Zuber V., Andreassen B.K., Witoelar A., Zeng L., Bettella F., Wang Y., McEvoy L.K., Thompson W.K., Schork A.J. (2016). Identifying Novel Gene Variants in Coronary Artery Disease and Shared Genes with Several Cardiovascular Risk Factors. Circ. Res..

[84] Ghosh S., Vivar J., Nelson C.P., Willenborg C., Segrè A.V., Mäkinen V.P., Nikpay M., Erdmann J., Blankenberg S., O’Donnell C. (2015). Systems Genetics Analysis of Genome-Wide Association Study Reveals Novel Associations Between Key Biological Processes and Coronary Artery Disease. Arteriosclerosis, thrombosis, and vascular biolog. Arter. Thromb. Vasc. Biol..

[85] Won H.H., Natarajan P., Dobbyn A., Jordan D.M., Roussos P., Lage K., Raychaudhuri S., Stahl E., Do R. (2015). Disproportionate Contributions of Select Genomic Compartments and Cell Types to Genetic Risk for Coronary Artery Disease. PLoS Genet..

[86] Blum C.B. (2016). Type III Hyperlipoproteinemia: Still Worth Considering?. Prog. Cardiovasc. Dis..

[87] Atis O., Sahin S., Ceyhan K., Ozyurt H., Akbas A., Benli I. (2016). The Distribution of Apolipoprotein E Gene Polymorphism and Apolipoprotein E Levels among Coronary Artery Patients Compared to Controls. Eurasian J. Med..

[88] Eichne J.E., Dunn S.T., Perveen G., Thompson D.M., Stewart K.E., Stroehla B.C. (2002). Apolipoprotein E polymorphism and cardiovascular disease: A HuGE review. Am. J. Epidemiol..

[89] Gerdes L.U., Jeune B., Ranberg K.A., Nybo H., Vaupel J.W. (2000). Estimation of apolipoprotein E genotype-specific relative mortality risks from the distribution of genotypes in centenarians and middle-aged men: Apolipoprotein E gene is a “frailty gene”, not a “longevity gene”. Genet. Epidemiol..

[90] Humphries S.E., Talmud P.J., Hawe E., Bolla M., Day I.N., Miller G.J. (2001). Apolipoprotein E4 and coronary heart disease in middle-aged men who smoke: A prospective study. Lancet.

[91] Talmud P.J., Lewis S.J., Hawe E., Martin S., Acharya J., Marmot M.G., Humphries S.E., Brunner E.J. (2004). No APOEepsilon4 effect on coronary heart disease risk in a cohort with low smoking prevalence: The Whitehall II study. Atherosclerosis.

[92] Chiodini B.D., Barlera S., Franzosi M.G., Beceiro V.L., Introna M., Tognoni G. (2003). APO B gene polymorphisms and coronary artery disease: A meta-analysis. Atherosclerosis.

[93] Hegele R.A., Huang L.S., Herbert P.N., Blum C.B., Buring J.E., Hennekens C.H., Breslow J.L. (1986). Apolipoprotein B-gene DNA polymorphisms associated with myocardial infarction. N. Engl. J. Med..

[94] Chen Y., Zeng J., Tan Y., Feng M., Qin J., Lin M., Zhao X., Zhao X., Liang Y., Zhang N. (2016). Association between apolipoprotein B EcoRI polymorphisms and coronary heart disease: A meta-analysis. Wien. Klin. Wochenschr..

[95] He K., Zhu Z., Chen Y. (2021). Lipoprotein Lipase Gene Polymorphisms Are Associated with Myocardial Infarction Risk: A Meta-Analysis. Genet. Test. Mol. Biomark..

[96] Ma W.Q., Wang Y., Han X.Q., Zhu Y., Liu N.F. (2018). Associations between LPL gene polymorphisms and coronary artery disease: Evidence based on an updated and cumulative meta-analysis. Biosci. Rep..

[97] Talmud P.J., Bujac S.R., Hall S., Miller G.J., Humphries S.E. (2000). Substitution of asparagine for aspartic acid at residue 9 (D9N) of lipoprotein lipase markedly augments risk of ischaemic heart disease in male smokers. Atherosclerosis.

[98] Chen Y.Q., Pottanat T.G., Zhen E.Y., Siegel R.W., Ehsani M., Qian Y.W., Konrad R.J. (2021). ApoA5 lowers triglyceride levels via suppression of ANGPTL3/8-mediated LPL inhibition. J. Lipid Res..

[99] Trabetti E., Biscuola M., Cavallari U., Malerba G., Girelli D., Olivieri O., Martinelli N., Corrocher R., Pignatti P.F. (2006). On the association of the oxidised LDL receptor 1 (OLR1) gene in patients with acute myocardial infarction or coronary artery disease. Eur. J. Hum. Genet..

[100] Salehipour P., Rezagholizadeh F., Mahdiannasser M., Kazerani R., Modarressi M.H. (2021). Association of OLR1 gene polymorphisms with the risk of coronary artery disease: A systematic review and meta-analysis. Heart Lung.

[101] Møller P.L., Rohde P.D., Winther S., Breining P., Nissen L., Nykjaer A., Bøttcher M., Nyegaard M., Kjolby M. (2021). Sortilin as a Biomarker for Cardiovascular Disease Revisited. Front. Cardiovasc. Med..

[102] Tsuzuki K., Itoh Y., Inoue Y., Hayashi H. (2019). TRB1 negatively regulates gluconeogenesis by suppressing the transcriptional activity of FOXO1. FEBS Lett..

[103] Douvris A., Soubeyrand S., Naing T., Martinuk A., Nikpay M., Williams A., Buick J., Yauk C., McPherson R. (2014). Functional analysis of the TRIB1 associated locus linked to plasma triglycerides and coronary artery disease. J. Am. Heart Assoc..

[104] Aimo A., Botto N., Vittorini S., Emdin M. (2019). Polymorphisms in the eNOS gene and the risk of coronary artery disease: Making the case for genome-wide association studies. Eur. J. Prev. Cardiol..

[105] Li X., Lin Y., Zhang R. (2019). Associations between endothelial nitric oxide synthase gene polymorphisms and the risk of coronary artery disease: A systematic review and meta-analysis of 132 case-control studies. Eur. J. Prev. Cardiol..

[106] Gholami M., Amoli M.M., Sharifi F., Khoshnevisan K. (2020). Comments on and assessments of ‘Associations between endothelial nitric oxide synthase gene polymorphisms and the risk of coronary artery disease: A systematic review and meta-analysis of 132 case-control studies. Eur. J. Prev. Cardiol..

[107] Wirka R.C., Wagh D., Paik D.T., Pjanic M., Nguyen T., Miller C.L., Kundu R., Nagao M., Coller J., Koyano T.K. (2019). Atheroprotective roles of smooth muscle cell phenotypic modulation and the TCF21 disease gene as revealed by single-cell analysis. Nat. Med..

[108] Zhao Q., Wirka R., Nguyen T., Nagao M., Cheng P., Miller C.L., Kim J.B., Pjanic M., Quertermous T. (2019). TCF21 and AP-1 interact through epigenetic modifications to regulate coronary artery disease gene expression. Genome Med..

[109] Pereira A., Palma Dos Reis R., Rodrigues R., Sousa A.C., Gomes S., Borges S., Ornelas I., Freitas A.I., Guerra G., Henriques E. (2016). Association of ADAMTS7 gene polymorphism with cardiovascular survival in coronary artery disease. Physiol. Genom..

[110] Aravani D., Morris G.E., Jones P.D., Tattersall H.K., Karamanavi E., Kaiser M.A., Kostogrys R.B., Ghaderi Najafabadi M., Andrews S.L., Nath M. (2019). HHIPL1, a Gene at the 14q32 Coronary Artery Disease Locus, Positively Regulates Hedgehog Signaling and Promotes Atherosclerosis. Circulation.

[111] Amara A., Mrad M., Sayeh A., Lahideb D., Layouni S., Haggui A., Fekih-Mrissa N., Haouala H., Nsiri B. (2018). The Effect of ACE I/D Polymorphisms Alone and with Concomitant Risk Factors on Coronary Artery Disease. Clin. Appl. Thromb. Hemost..

[112] Lindpaintner K., Lee M., Larson M.G., Rao V.S., Pfeffer M.A., Ordovas J.M., Schaefer E.J., Wilson A.F., Wilson P.W., Vasan R.S. (1996). Absence of association or genetic linkage between the angiotensin-converting-enzyme gene and left ventricular mass. N. Engl. J. Med..

[113] Keavney B., McKenzie C., Parish S., Palmer A., Clark S., Youngman L., Delépine M., Lathrop M., Peto R., Collins R. (2000). Large-scale test of hypothesised associations between the angiotensin-converting-enzyme insertion/deletion polymorphism and myocardial infarction in about 5000 cases and 6000 controls. International Studies of Infarct Survival (ISIS) Collaborators. Lancet.

[114] Borai I.H., Hassan N.S., Shaker O.G., Ashour E.E., Badrawy M.E.I., Olfat M., Fawzi L., Mageed L. (2018). Synergistic effect of ACE and AGT genes in coronary artery disease. J. Basic Appl. Sci..

[115] Feng X., Zheng B.S., Shi J.J., Qian J., He W., Zhou H.F. (2014). A systematic review and meta-analysis of the association between angiotensin II type 1 receptor A1166C gene polymorphism and myocardial infarction susceptibility. J. Renin-Angiotensin-Aldosterone Syst..

[116] Liu D.X., Zhang Y.Q., Hu B., Zhang J., Zhao Q. (2015). Association of AT1R polymorphism with hypertension risk: An update meta-analysis based on 28,952 subjects. J. Renin-Angiotensin-Aldosterone Syst..

[117] Sun J., Zhao M., Miao S., Xi B. (2016). Polymorphisms of three genes (ACE, AGT and CYP11B2) in the renin-angiotensinaldosterone system are not associated with blood pressure salt sensitivity: A systematic meta-analysis. Blood Press..

[118] Wang L., Zhang Z., Liu D., Yuan K., Zhu G., Qi X. (2020). Association of -344C/T polymorphism in the aldosterone synthase (CYP11B2) gene with cardiac and cerebrovascular events in Chinese patients with hypertension. J. Int. Med. Res..

[119] Hautanen A., Toivanen P., Mänttäri M., Tenkanen L., Kupari M., Manninen V., Kayes K.M., Rosenfeld S., White P.C. (1999). Joint effects of an aldosterone synthase (CYP11B2) gene polymorphism and classic risk factors on risk of myocardial infarction. Circulation.

[120] Kroll H., Gardemann A., Fechter A., Haberbosch W., Santoso S. (2000). The impact of the glycoprotein Ia collagen receptor subunit A1648G gene polymorphism on coronary artery disease and acute myocardial infarction. Thromb. Haemost..

[121] Santoso S., Kunicki T.J., Kroll H., Haberbosch W., Gardemann A. (1999). Association of the platelet glycoprotein Ia C807T gene polymorphism with nonfatal myocardial infarction in younger patients. Blood.

[122] Reiner A.P., Schwartz S.M., Kumar P.N., Rosendaal F.R., Pearce R.M., Aramaki K.M., Psaty B.M., Siscovick D.S. (2001). Platelet glycoprotein IIb polymorphism, traditional risk factors and non-fatal myocardial infarction in young women. Br. J. Haematol..

[123] Hato T., Minamoto Y., Fukuyama T., Fujit S. (1997). Polymorphisms of HPA-1 through 6 on platelet membrane glycoprotein receptors are not a genetic risk factor for myocardial infarction in the Japanese population. Am. J. Cardiol..

[124] Floyd C.N., Mustafa A., Ferro A. (2014). The PlA1/A2 polymorphism of glycoprotein IIIa as a risk factor for myocardial infarction: A meta-analysis. PLoS ONE.

[125] Bojesen S.E., Juul K., Schnohr P., Tybjaerg-Hansen A., Nordestgaard B.G. (2003). Copenhagen City Heart Study. Platelet glycoprotein IIb/IIIa Pl(A2)/Pl(A2) homozygosity associated with risk of ischemic cardiovascular disease and myocardial infarction in young men: The Copenhagen City Heart Study. J. Am. Coll. Cardiol..

[126] Liang Z., Jiang W., Ouyang M., Yang K. (2015). PAI-1 4G/5G polymorphism and coronary artery disease risk: A meta-analysis. Int. J. Clin. Exp. Med..

[127] Khaki-Khatibi F., Karimian A. (2018). Association of PAI-1 serum levels and polymorphism of gene of Plasminogen Activator Inhibitor-1 in patient of Non-diabetic and Non-smoker with Coronary Artery Disease. Med. J. Tabriz Univ. Med. Sci..

[128] Zhang X.J., Wei C.Y., Li W.B., Zhang L.L., Zhou Y., Wang Z.H., Tang M.X., Zhang W., Zhang Y., Zhong M. (2015). Association between single nucleotide polymorphisms in thrombospondins genes and coronary artery disease: A meta-analysis. Thromb. Res..

[129] Ercan B., Tamer L., Sucu N., Pekdemir H., Camsari A., Atik U. (2008). Factor VLeiden and prothrombin G20210A gene polymorphisms in patients with coronary artery disease. Yonsei Med. J..

[130] Lewis S.J., Ebrahim S., Smith G.D. (2005). Meta-analysis of MTHFR 677C->T polymorphism and coronary heart disease: Does totality of evidence support causal role for homocysteine and preventive potential of folate?. BMJ.

[131] Brattström L., Wilcken D.E., Ohrvik J., Brudin L. (1998). Common methylenetetrahydrofolate reductase gene mutation leads to hyperhomocysteinemia but not to vascular disease: The result of a meta-analysis. Circulation.

[132] Nedelcu C., Ionescu M., Pantea-Stoian A., Niţă D., Petcu L., Mazilu L., Suceveanu A.I., Tuţă L.A., Parepa I.R. (2021). Correlation between plasma homocysteine and first myocardial infarction in young patients: Case-control study in Constanta County, Romania. Exp. Ther. Med..

[133] Klerk M., Verhoef P., Clarke R., Blom H.J., Kok F.J., Schouten E.G. (2002). MTHFR Studies Collaboration Group. MTHFR 677C-->T polymorphism and risk of coronary heart disease: A meta-analysis. JAMA.

[134] Kelberman D., Hawe E., Luong L.A., Mohamed-Ali V., Lundman P., Tornvall P., Aillaud M.F., Juhan-Vague I., Yudkin J.S., Margaglione M. (2004). HIFMECH study group. Effect of Interleukin-6 promoter polymorphisms in survivors of myocardial infarction and matched controls in the North and South of Europe. The HIFMECH Study. Thromb. Haemost..

[135] Gudmundsson G., Matthiasson S.E., Arason H., Johannsson H., Runarsson F., Bjarnason H., Helgadottir K., Thorisdottir S., Ingadottir G., Lindpaintner K. (2002). Localization of a gene for peripheral arterial occlusive disease to chromosome 1p31. Am. J. Hum. Genet..

[136] Pajukanta P., Cargill M., Viitanen L., Nuotio I., Kareinen A., Perola M., Terwilliger J.D., Kempas E., Daly M., Lilja H. (2000). Two loci on chromosomes 2 and X for premature coronary heart disease identified in early- and late-settlement populations of Finland. Am. J. Hum. Genet..

[137] Harrap S.B., Zammit K.S., Wong Z.Y., Williams F.M., Bahlo M., Tonkin A.M., Anderson S.T. (2002). Genome-wide linkage analysis of the acute coronary syndrome suggests a locus on chromosome 2. Arter. Thromb. Vasc. Biol..

[138] Lange L.A., Lange E.M., Bielak L.F., Langefeld D., Kardia S.L., Royston P., Turner S.T., Sheedy P.F., Boerwinkle E., Peyser P.A. (2002). Autosomal genome-wide scan for coronary artery calcification loci in sibships at high risk for hypertension. Arter. Thromb. Vasc. Biol..

[139] Zhu L.P., Yin W.L., Peng L., Zhou X.H., Zhou P., Xuan S.X., Luo Y., Chen C., Cheng B., Lin J.D. (2021). Association of Aldehyde Dehydrogenase 2 Gene Polymorphism with Myocardial Infarction. J. Inflamm. Res..

[140] Stevens H.Y., Melchior B., Bell K.S., Yun S., Yeh J.C., Frangos J.A. (2008). PECAM-1 is a critical mediator of atherosclerosis. Dis. Model. Mech..

[141] Thomas C.B., Cohen B.H. (1955). The familial occurrence of hypertension and coronary artery disease, with observations concerning obesity and diabetes. Ann. Intern. Med..

[142] Brown D., Giles W.H., Burke W., Greenlund K.J., Croft J.B. (2002). Familial aggregation of early-onset myocardial infarction. Community Genet..

[143] Schildkraut J.M., Myers R.H., Cupples L.A., Kiely D.K., Kannel W.B. (1989). Coronary risk associated with age and sex of parental heart disease in the Framingham Study. Am. J. Cardiol..

[144] Chacko M., Sarma P.S., Harikrishnan S., Zachariah G., Jeemon P. (2020). Family history of cardiovascular disease and risk of premature coronary heart disease: A matched case-control study. Wellcome Open Res..

[145] Zdravkovic S., Wienke A., Pedersen N.L., Marenberg M.E., Yashin A.I., De Faire U. (2002). Heritability of death from coronary heart disease: A 36-year follow-up of 20966 Swedish twins. J. Intern. Med..

[146] Wienke A., Holm N.V., Skytthe A., Yashin A.I. (2001). The heritability of mortality due to heart diseases: A correlated frailty model applied to Danish twins. Twin Res..

[147] Jansen H., Samani N.J., Schunkert H. (2014). Mendelian randomization studies in coronary artery disease. Eur. Heart J..

[148] Kjeldsen E.W., Thomassen J.Q., Frikke-Schmidt R. (2022). HDL cholesterol concentrations and risk of atherosclerotic cardiovascular disease—Insights from randomized clinical trials and human genetics. Biochim. Biophys. Acta Mol. Cell Biol. Lipids.

[149] Lewis C.M., Vassos E. (2020). Polygenic risk scores: From research tools to clinical instruments. Genome Med..

[150] Klarin D., Natarajanm P. (2021). Clinical utility of polygenic risk scores for coronary artery disease. Nat. Rev. Cardiol..

